# Evaluating the sampling effort for the metabarcoding‐based detection of fish environmental DNA in the open ocean

**DOI:** 10.1002/ece3.9921

**Published:** 2023-03-24

**Authors:** Tatsuya Kawakami, Aya Yamazaki, Maki Asami, Yuko Goto, Hiroki Yamanaka, Susumu Hyodo, Hiromichi Ueno, Akihide Kasai

**Affiliations:** ^1^ Faculty of Fisheries Sciences Hokkaido University Hakodate Hokkaido Japan; ^2^ Research and Educational Unit for Studies on Connectivity of Hills, Humans and Oceans Kyoto University Kyoto Japan; ^3^ Center for Biodiversity Science Ryukoku University Otsu Shiga Japan; ^4^ Faculty of Advanced Science and Technology Ryukoku University Otsu Shiga Japan; ^5^ Atmosphere and Ocean Research Institute, The University of Tokyo Kashiwa Chiba Japan

**Keywords:** asymptotic analysis, Chukchi Sea, dissimilarity, northwestern Pacific Ocean, replicated sampling

## Abstract

Clarifying the effect of the sampling protocol on the detection of environmental DNA (eDNA) is essential for appropriately designing biodiversity research. However, technical issues influencing eDNA detection in the open ocean, which consists of water masses with varying environmental conditions, have not been thoroughly investigated. This study evaluated the sampling effort for the metabarcoding‐based detection of fish eDNA using replicate sampling with filters of different pore sizes (0.22 and 0.45 μm) in the subtropical and subarctic northwestern Pacific Ocean and Arctic Chukchi Sea. The asymptotic analysis predicted that the accumulation curves for detected taxa did not saturate in most cases, indicating that our sampling effort (7 or 8 replicates, corresponding to 10.5–40 L of filtration in total) was insufficient to fully assess the species diversity in the open ocean and that tens of replicates or a substantial filtration volume were required. The Jaccard dissimilarities between filtration replicates were comparable with those between the filter types at any site. In subtropical and subarctic sites, turnover dominated the dissimilarity, suggesting that the filter pore size had a negligible effect. In contrast, nestedness dominated the dissimilarity in the Chukchi Sea, implying that the 0.22 μm filter could collect a broader range of eDNA than the 0.45 μm filter. Therefore, the effect of filter selection on the collection of fish eDNA likely varies depending on the region. These findings highlight the highly stochastic nature of fish eDNA collection in the open ocean and the difficulty of standardizing the sampling protocol across various water masses.

## Graphical abstract

We evaluated the sampling effort for the metabarcoding‐based detection of fish eDNA in the subtropical and subarctic northwestern Pacific Ocean and Arctic Chukchi Sea. The results suggested that tens of replicates or a substantial filtration volume were required to fully describe the species diversity in the open ocean, and in most cases, the turnover component predominated the compositional dissimilarities between filters and even within the filtration replicates. These findings highlight the highly stochastic nature of fish eDNA collection in the open ocean.

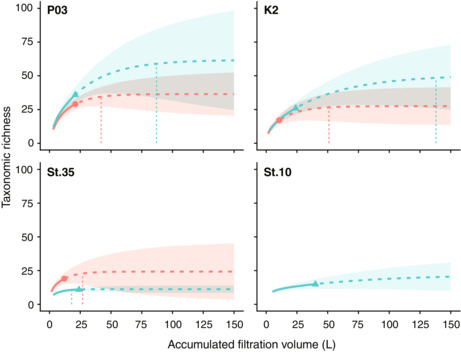

## 1 INTRODUCTION

Recent global climate change has induced fish diversity losses and range shifts in the ocean (Campana et al., 2020; Sunday et al., 2012; Worm et al., 2005). Because fish are an essential component of the marine food web, linking primary production and organisms at higher trophic levels, their redistribution can alter the food web structure (Kortsch et al., 2015). Furthermore, it is expected that altered fish distribution patterns lead to social and economic changes through the impact on subsistence and commercial fisheries (Carothers et al., 2019; Fauchald et al., 2021). Continuously monitoring fish fauna across broad spatial and temporal ranges is important for predicting how the marine ecosystem and human society respond to ongoing climate change.

The paucity of reliable baseline data on fish distribution and abundance hinders the evaluation of long‐term ecological changes (Wassmann et al., 2011). In the open ocean, arranging comparable datasets for examining fish diversity is a challenge because of difficulties in accessibility and limited research opportunities. Traditionally, the investigation of fish diversity and distribution in the open ocean has relied on fishery catchment data or large‐scale fishery‐independent surveys, which are time‐consuming and expensive (Fossheim et al., 2015; Hansen et al., 2018; Worm et al., 2005). Fisheries and ship‐borne surveys often employ net sampling (bottom trawl and purse seine net) and pelagic longlines, which can be highly destructive (Fossheim et al., 2015; Worm et al., 2005). These methods can also produce biased data due to gear avoidance and the selective performance of fishing operations (Clegg et al.,  2022). Additionally, catchment data focus on target species, making it challenging to perform community‐level analyses. Therefore, a novel method that requires less effort and is nondestructive is necessary to collect more comprehensive and less biased data.

Environmental DNA (eDNA) metabarcoding is a promising method for replacing or complementing conventional methods for biodiversity monitoring (Deiner et al., 2017). Because eDNA metabarcoding can simultaneously obtain eDNA from a wide variety of taxa in an environmental sample, it has increasingly been used to describe fish diversity and distribution in the ocean, from coastal areas to tropical and subarctic regions (Afzali et al., 2021; Fraija‐Fernández et al., 2020; Jeunen, Knapp, Spencer, Lamare, et al., 2019; Larson et al., 2022; Oka et al., 2021; Sigsgaard et al., 2017; Stat et al., 2019; Yamamoto et al., 2017), with a few open ocean studies (Canals et al., 2021; Sigsgaard et al., 2019; Thomsen et al., 2016). These studies indicated that eDNA analysis could produce results comparable with those of conventional methods and sometimes outperform them in estimating species richness (Sigsgaard et al., 2017, 2019; Thomsen et al., 2012, 2016; Yamamoto et al., 2017).

While eDNA metabarcoding has successfully been applied in various environments, many technical issues influencing the detection of eDNA have been raised (Deiner et al., 2017; Goldberg et al., 2016). Thus, pilot studies are recommended to optimize the sampling protocol for each application. Unlike conventional methods, eDNA indirectly indicates the presence of fish species by detecting DNA that originates from an individual fish and undergoes degradation under the influence of various biotic and abiotic environmental factors and dispersion from their source by currents or vertical mixing and particle sinking (Barnes & Turner, 2016). Consequently, fish eDNA in the open ocean is thought to represent a small fraction of DNA (Hansen et al., 2018; Stat et al., 2017). This finding implies that without sufficient sampling, eDNA analysis in the open ocean is prone to underestimating species richness owing to false‐negative errors. Additionally, environmental conditions that influence eDNA persistence, such as pH, water temperature, UV intensity, and the presence of polymerase chain reaction (PCR) inhibitors (Andruszkiewicz et al., 2017; Collins et al., 2018; Jo et al., 2019; Stoeckle et al., 2017), are heterogeneous in the open ocean. Because optimization efforts of the sampling method for detecting fish eDNA have focused on coastal (Bessey et al., 2020; Stauffer et al., 2021) and freshwater environments (Cantera et al., 2019), further efforts are required in the open ocean.

One way to improve the detectability of fish eDNA is to increase filtration volume (Cantera et al., 2019). Previous fish eDNA surveys in the ocean often filtered 0.5–2 L of seawater per sample (e.g., Afzali et al., 2021; Sigsgaard et al., 2017; Thomsen et al., 2016) or 5 L using prefilter to avoid clogging (Canals et al., 2021; Fraija‐Fernández et al., 2020). These studies obtained sufficient species richness to depict seasonality and differences among habitats in the fish community. However, recent studies have suggested that filtering tens or hundreds of liters of water is required to completely detect the expected fish fauna at a site (Bessey et al., 2020; Bylemans et al., 2018; Cantera et al., 2019; Stauffer et al., 2021). However, although increasing the filtration volume per filter would certainly increase the probability of collecting rare eDNA, filtration of a massive amount of water through a filter with a small pore size (0.2–0.7 μm for fish eDNA surveys) is unrealistic due to filter clogging, labor intensity, and the high cost of filters. Therefore, making sampling replicates at a single site is a plausible way to increase the total filtration volume and thus improve fish eDNA detection.

Enhancing the efficacy of eDNA capture with an appropriate filter is another possible approach for improving eDNA detectability in metabarcoding (Deiner et al., 2018). Experimental studies have indicated a strong influence of both filter material and pore size on the maximum filtration volume, total DNA yield, estimation of species richness, and detection probability of fish eDNA (Deiner et al., 2018; Jeunen, Knapp, Spencer, Taylor, et al., 2019; Minamoto et al., 2016; Spens et al., 2017). After reports on the advantage of using Sterivex filter units (Millipore, Burlington, MA, USA) to capture eDNA (Li et al., 2018; Spens et al., 2017), Sterivex 0.45 μm filters have been often employed in studies focusing on fish eDNA (e.g., Canals et al., 2021; Fraija‐Fernández et al., 2020; Oka et al., 2021). Recent studies focusing on particle size distribution (PSD) of fish eDNA have shown that fish mitochondrial eDNA are primarily found as particles of 1–10 μm in size (Barnes et al., 2021; Cooper et al., 2022; Jo et al., 2019; Zhao et al., 2021). These results indicated that 0.45 μm filters are appropriate for collecting fish eDNA. However, various environmental factors complicate eDNA persistence (Jo & Minamoto, 2021), and it is unclear whether the standard filtering method is appropriate for fish eDNA detection in the open ocean. Particles containing fish eDNA shift their size distribution toward a smaller size fraction with increased time after release (Jo et al., 2019). This implies that degraded eDNA with a smaller size fraction can be detected at a distance from the source organism due to dilution and transportation. In this case, using a 0.22 μm filter may improve the completeness of fish eDNA detection because eDNA present only in the 0.22–0.45 μm fraction could provide information that would complement the result obtained from the over 0.45 μm fraction.

This study aimed to evaluate how filtration volume and pore size affect the detection of fish species using eDNA metabarcoding. To achieve this aim, replicated sampling was performed at sites far from the land, from the subtropical and subarctic northwestern Pacific Ocean to the Arctic Chukchi Sea. Subsequently, the effects of filtration volume and filter pore size were evaluated based on asymptotic analysis and dissimilarity index, as used in previous studies (e.g., Stauffer et al., 2021). Based on the results, we discuss how eDNA sampling strategies can be optimized to estimate fish biodiversity in the open ocean.

## 2 MATERIALS AND METHODS

### 2.1 Study sites

**FIGURE 1:**
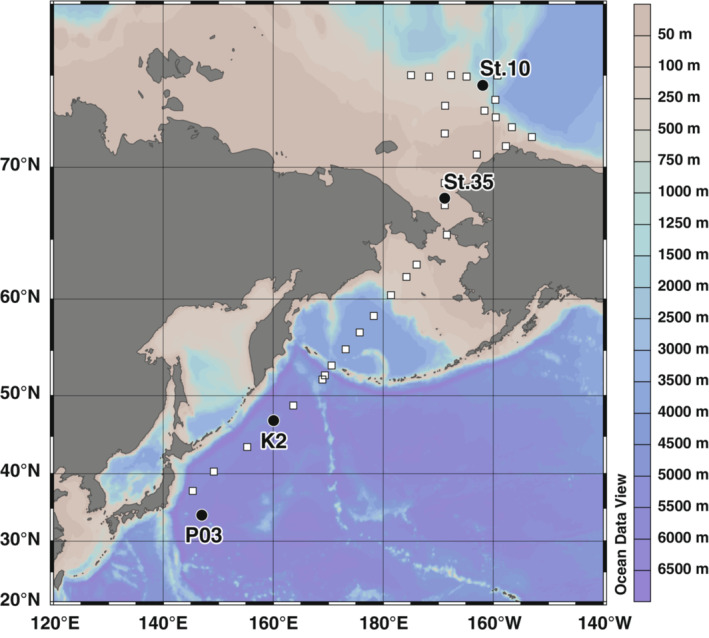
Map showing the sampling sites for this study. The black circles represent eDNA sampling sites where filtration replicates were created using two different types of filters (022_Sterivex: 0.22 μm Sterivex‐GV and 045_Sterivex: 0.45 μm Sterivex‐HV) to assess the effect of sampling protocol on fish eDNA detection. The white squares represent the sites where the maximum filtration volume until clogging and filtrate speed were measured during the daily eDNA collection.

Sampling of eDNA was performed at sites in the subtropical (P03) and subarctic (K2) gyres of the northwestern Pacific Ocean and the shelf (St. 35) and slope (St. 10) of the Chukchi Sea during a cruise (MR20‐05C) conducted by the “R/V Mirai” of the Japan Agency for Marine‐Earth Science and Technology, from September 19 to November 2, 2020 (Figure 1). Temperature (°C), salinity, dissolved oxygen (μmol/kg), chlorophyll‐*a* (chl‐*a*) fluorescence (RFU), and turbidity (NTU) in surface water at the sites were obtained from the Continuous Sea Surface Water Monitoring System (Marine Works Japan Co., Ltd., Kanagawa, Japan). These measurements clearly indicated that the four sites had different environmental conditions (Table 1). The current speed (m/s) was obtained from the hull‐mounted acoustic Doppler current profiler (Teledyne RD Instruments, CA, USA).

**TABLE 1:** Coordinates of the study sites where filtration replicates were created using two different types of filters (022_Sterivex: 0.22 μm Sterivex‐GV and 045_Sterivex: 0.45 μm Sterivex‐HV) for evaluating the effect of sampling protocol on fish eDNA detection. The eDNA sampling was conducted during the cruise of the R/V Mirai (MR20‐05C), and environmental conditions at the study sites were obtained from the onboard monitoring system.

Station	Description	Date	Latitude	Longitude	Bottom depth (m)	Water temperature (°C)	Salinity	Chl *a* fluorescence (RFU)	Turbidity (NTU)	Dissolved oxygen (μmol/kg)	Current speed (m/s)
St.10	Chukchi Sea slope	October 10, 2020	74°31.587 N	161°55.609 W	1690	0.0	26.4	1.55	7.52	403.23	0.2
St.35	Chukchi Sea shelf	October 21, 2020	68°00.004 N	168°49.989 W	57.5	3.1	32.3	1.52	27.32	330.1	0.2
K2	Subarctic gyre	November 1, 2020	47°00.044 N	160°02.042 E	5233	11.9	32.7	8.37	14.52	290.38	0.3
P03	Subtropical gyre	September 23, 2020	34°00.047 N	146°59.842 E	5831	28.8	34.4	0.48	11.96	194.52	0.1

### 2.2 Sampling design

To investigate the effect of filtration volume and pore size on fish eDNA detection by metabarcoding, two types of Sterivex filter units (capacity: 2.2 mL, filtration area: 10 cm^2^), with different pore sizes (0.22 μm [Sterivex‐GV] and 0.45 μm [Sterivex‐HV], hereafter named 022_Sterivex and 045_Sterivex, respectively) but consisting of the same material (polyvinylidene difluoride), were used for the replicated sampling. At St. 10, only 045_Sterivex was tested. Seven or eight filtration replicates were performed for each site (Table S1). Because of its widespread use in eDNA studies and the availability of a simplified standard protocol for DNA extraction (Minamoto et al., 2020; Wong et al., 2020), the Sterivex filter was chosen to evaluate the sampling effort to make this study's findings applicable to future research. Although a high‐capacity capsule filter with 0.45 μm pore size can filter over 30 L of water per filter without clogging (e.g., Stauffer et al., 2021), such a filter was not evaluated because the DNA extraction protocol using this type of filter is more complicated and time‐consuming owing to requirements of resuspension and precipitation processes (Valentini et al., 2016). In addition, there is a potential problem in the collection and transportation of large amounts of water.

Standardizing the filtration volume is crucial to reasonably compare the resultant taxonomic composition among filtration replicates. The suitable amount of water per replicate can be defined as the amount that collects sufficient yield of fish eDNA for metabarcoding analysis without unexpected clogging of the filter. Previous studies have often filtered 0.5–2 L of seawater with a single Sterivex to detect fish eDNA, regardless of its pore size (e.g., Oka et al., 2021; Sigsgaard et al., 2017). However, there was no prior knowledge of the suitable filtration volume at our study sites. Therefore, the maximum filtration volume of 022_Sterivex was examined in the eDNA sampling at P03, and the standard filtration volume per replicate was accordingly determined as 3 L for both pore sizes. However, the filtration volume was reduced to 1.5 L per replicate for 022_Sterivex at K2 and St. 35 because the filtration speed decreased drastically as filtration progressed. This volume was intended to reasonably compare the results between the filters at the same pooled filtration volume. Meanwhile, 5 L of seawater was filtered using a 045_Sterivex at St. 10 because a low yield of fish eDNA was expected in the vicinity of the sea ice. The results from one filtration replicate at St. 35 using 045_Sterivex were excluded from the analysis because of an unintended filtration volume.

In addition, the effort and time required to collect eDNA in the open ocean were evaluated based on the information obtained from daily eDNA sampling for fish community analysis from the Northwestern Pacific to the Arctic region, which was conducted independently of the eDNA sampling for this study during the same cruise (Figure 1). During the daily sampling, the maximum filtration volume (when the filter was clogged) and average filtrate speed were recorded.

### 2.3 Contamination control during eDNA sampling and molecular work

All water collection and filtration procedures were performed in the ship's laboratory, where no organism samples were processed. To minimize the risk of cross‐contamination, all equipment used for water collection and filtration were decontaminated by soaking in a 2% bleach solution (approximately 0.12% NaOCl) for at least 10 min. The equipment were then rinsed with tap water and distilled water to remove residual bleach. The workspace was wiped using bleach disinfectant spray (approximately 0.1% NaOCl). New gloves and masks were used for water collection and filtration. Negative controls were created for every site and filter type by filtering 500 mL of Milli‐Q water, following the same procedure as that for the seawater sampling.

For DNA extraction and PCR preparation, samples were processed in a laboratory dedicated to eDNA analysis and transferred to another room for PCR, ensuring that no tissue or PCR product was handled. PCR amplification and library preparation were performed in a separate room, as was sequencing. Researchers always wore new gloves and masks for every step of the molecular work. The workspace and all equipment (micropipettes, incubator, and centrifuge) were wiped with DNA‐OFF (Takara, Shiga, Japan) and distilled water before use. In addition, the extraction equipment (manifold, valves, and tubes) were decontaminated using bleach solution, as described above.

### 2.4 eDNA sampling

For eDNA sampling, seawater pumped from approximately 4.5 m depth was collected from the faucet of the ship's laboratory. A recent study indicated that fish eDNA is vertically stratified along the water column, reflecting the vertical distribution of fish (Canals et al., 2021). Vertical differences in eDNA detection can be observed even between the surface water and 50 m depth (Canals et al., 2021). Therefore, the fish eDNA detected in this study could be considered as eDNA that originated from pelagic fish inhabiting surface water.

Seawater was collected when the ship stopped to conduct conductivity–temperature–depth (CTD) observations to ensure that the filtration replicates were taken from the same site. Because the seawater was kept constantly flowing (approximately 5 L/min), the inside of the derivation pipes and tubes was always rinsed with fresh seawater. At each site, water samples were collected in four or eight 10‐L plastic bags. Benzalkonium chloride solution (BAC, final concentration of 0.01%) was added to each bag to prevent eDNA degradation (Jo et al., 2021). The fixed amount of seawater described above was then dispensed to other 10‐L plastic bags. Before dispensing the water, the bags were gently mixed to minimize heterogeneity due to particle sedimentation. Each bag was assigned one or two filtration replicates of 045_Sterivex and 022_Sterivex. Prior to collecting or dispensing the seawater, the bags were rinsed with a small amount of seawater three times and filled with seawater to reduce the possible risk of DNA loss by absorbance in plastic.

Water samples were vacuum‐filtered onto the filters using an aspirator (GAS‐1 N; AS ONE, Osaka, Japan) connected to a manifold (Multivac 310‐MS; Rocker, Kaohsiung City, Taiwan). A Sterivex filter was installed between the sample bag and manifold using homemade adapters consisting of vacuum tubing and luer fittings. The pressure was adjusted from −0.08 to −0.06 MPa to avoid air binding and mechanical damage to the filter. This pressure equilibrated the flow rates at the inlet and outlet of the Sterivex, preventing the filter from being exposed to a vacuum. After filtration, the filter was immersed in 1.6 mL of RNA*later* (Thermo Fisher Scientific, Waltham, MA, USA) and stored at −20°C until DNA extraction (Minamoto et al., 2020). For daily eDNA sampling, the seawater was processed following the same procedure as the replicated sampling, but without adding BAC, and filtration was continued until clogging became severe.

### 2.5 DNA extraction

DNA was individually extracted from each filtration replicate using the Qiagen DNeasy Blood and Tissue kit (Qiagen, Hilden, Germany), following Wong et al. (2020), in which the original extraction protocol was modified to maximize the DNA yield from the Sterivex filter. First, RNA*later* was removed by vacuum filtration using a manifold (QIAvac; Qiagen) and vacuum pump (MAS‐01; AS ONE). A VacValve (Qiagen) was used in combination with the manifold throughout the extraction to decrease the contamination risk from flowback. A lysis buffer mix (PBS [990 μL], Buffer AL [910 μL], Proteinase K [100 μL]; 2 mL total volume) was introduced through the outlet using a 2.5‐mL syringe (Terumo Corporation, Tokyo, Japan). The filter was sealed with luer fittings and incubated at 56°C for 30 min, without rotation.

The lysate was retrieved in a 3‐mL tube attached to the filter inlet by centrifugation at 2500 ***g*** for 10 min. Ethanol (99.5%; FUJIFILM Wako Pure Chemical Corporation, Osaka, Japan) was added to the lysate at one‐third of the final volume (1 mL). The mixture was loaded and filtered on a DNeasy Blood and Tissue kit column attached to the manifold. The column was washed using 0.8 mL of AW1 buffer and 0.8 mL of AW2 buffer sequentially and then dried via centrifugation at 21,300 ***g*** for 2 min.

The adsorbed DNA was eluted from the column into a DNA LoBind Tube (Eppendorf, Hamburg, Germany) twice with 75 μL of AE buffer to maximize recovery. Samples (150 μL) were collected for each extraction. The extracts were stored at −20°C.

### 2.6 PCR and sequencing

The partial mitochondrial 12S rRNA gene (approximately 170 bp) contained in the DNA extracts from each filtration replicate was amplified using MiFish primers (Miya et al., 2015) and sequenced using the Illumina paired‐end index sequencing protocol. Although there is a limitation in species‐level discrimination for some fish taxa when using the 12S rRNA gene as a marker for species identification (Cawthorn et al., 2012; Miya et al., 2015), MiFish primers have frequently been used for fish eDNA metabarcoding. A recent study recommended using MiFish primers for fish eDNA metabarcoding because they show better performance for marine fish detection in terms of specificity, discriminatory power, and reproducibility than other universal primers used for the DNA metabarcoding of fish (Collins et al., 2019). Another limitation is that the MiFish region has much lower species coverage of the reference database for marine fish than other primer pairs targeting COI or 16S rRNA genes (Marques et al., 2021). However, even though there is a paucity of reference data, representative sequences can at least be distinguished as a molecular operational taxonomic unit (MOTU; Floyd et al., 2002) if the primer pairs have sufficient discriminatory power. Therefore, it is predicted that using MiFish primers would provide the most comprehensive view of fish diversity in the open ocean.

The first PCR amplification of the target region was performed using eight technical replicates for each sample. MiFish‐U and its variant, MiFish‐E, which improves the universality of Elasmobranchii, were used to cover a broader taxonomic range (Minamoto et al., 2020). The 20‐μL reaction contained 1× PCR Buffer for KOD FX Neo (Toyobo, Osaka, Japan), 0.4 mM dNTP mix, 0.4 U KOD FX Neo polymerase, 0.29 μM of each MiFish‐U primer, 0.15 μM of each MiFish‐E primer, and 2 μL of the template. The PCR conditions were as follows: 94°C for 2 min; 40 cycles of 98°C for 10 s, 65°C for 30 s, and 68°C for 30 s; followed by a final extension at 68°C for 5 min. A no‐template control (NTC) was introduced in every run of the first PCR to evaluate cross‐contamination during PCR and sequencing with 2.0 μL of nuclease‐free water (UltraPure; Invitrogen, Waltham, MA, USA) in place of extracted DNA. Individual PCR replicates were pooled to minimize PCR dropouts and purified using ExoSAP‐IT (Thermo Fisher Scientific). As a result, the first PCR produced DNA fragments of approximately 300 bp containing the target region of metabarcoding franked by binding sites for sequencing primers.

A second PCR was performed to attach the Illumina sequence adapter and dual‐index tags (i7 and i5) to the products of the first PCR. The second PCR product for each sample contained a unique combination of tags. The reaction volume was 13 μL, containing 1× PCR Buffer for KOD FX Neo, 0.37 mM dNTP mix, 0.24 U KOD FX Neo polymerase, 0.27 μM of each index primer, and 2 μL of the first PCR product. The second PCR products were pooled in one tube with a volume of 2–6 μL for the sample, for which the volume was determined based on band brightness after gel electrophoresis, and 4 μL for the NTC and negative controls. The concentrations of the PCR products were not normalized because the negative effect of normalization on the detected biodiversity when a single dominant species overwhelms the sample has been documented (Deagle et al., 2018). The pooled library was purified using AMPure XP (Beckman Coulter, Brea, CA, USA). The second PCR products with a length of approximately 370 bp were excised using 2% E‐Gel SizeSelect Agarose Gel (Thermo Fisher Scientific) and quantified using a Qubit dsDNA HS Assay Kit and Qubit 4 Fluorometer (Thermo Fisher Scientific). Its purity was evaluated using a Qsep1 Bio‐Fragment Analyzer (BiOptic, Taipei, Taiwan) or an Agilent 2100 BioAnalyzer (Agilent, Santa Clara, CA, USA). The library was diluted to 40 pM with 10 mM Tris–HCl buffer (pH 8.5) or Buffer EB (Qiagen) and sequenced with an iSeq 100 (Illumina, San Diego, CA, USA) using the 150‐bp paired‐end reading with 30% PhiX spike‐in (Illumina) for quality control.

Although the length of our second PCR products was approximately 370 bp, only the internal part franked by two sequencing priming sites was sequenced (about 240 bp). In 150‐bp paired‐end reads, the sequencing reaction begins at the sequencing priming sites, and 150 cycles of reaction was carried out for each direction (forward and reverse). Thus, both strands can be combined into a single sequence by aligning their overlapping parts (about 50–60 bp).

The sequencing data used in this study were obtained from three iSeq runs. A set of filtration replicates and a negative control obtained from the same experimental conditions (site and filter type) were included in each single run (Table S1).

### 2.7 Processing sequence data

The raw sequences were demultiplexed using a built‐in module in iSeq 100 and exported as fastq files. The fastq files from three iSeq runs were pooled and processed using the Claident (v.0.9.2020.12.17; Tanabe & Toju, 2013; software available online: https://www.claident.org/) pipeline with default parameters. In this pipeline, the forward and reverse reads were merged after removing the primer sequences, and short (<100 bp) and low‐quality sequences (maximum number of expected errors ≥2) were removed. The amplicon sequence variants (ASVs) were constructed by denoising the quality‐filtered sequences using the DADA2 algorithm (Callahan et al., 2016). The chimeras were identified and removed before assembling the sample‐by‐ASV table for analysis.

All subsequent data handling and statistical analyses were performed using R v.4.1.2 (R Core Team, 2021) in combination with RStudio v.2022.02.3 (available online: https://www.rstudio.com/) and the packages implemented in it.

Possible contaminant reads were removed from each sample based on the sequencing results of the negative controls (field blank and NTCs). Because the relationship between the DNA concentration in the PCR products and the read count obtained from sequencing cannot be assumed to be comparable across different runs, this process was performed for each iSeq run. First, the mean read count per ASV in the negative controls was used as the cutoff value (8–13 reads). The ASVs detected in samples with read counts below this value were excluded. Second, any ASV found in the sample was considered positive when the read count exceeded the maximum read count of each ASV detected in all negative controls within a single run. Although it has been reported that the patterned flow cell implemented in iSeq 100 generates misassignment between samples with higher frequency than the nonpatterned flow cell (0.10%–0.29%, MacConaill et al., 2018), these thresholds could effectively reduce the risk of false‐positive detection. Before applying these detection thresholds, the read count in each ASV was corrected in proportion to the volume of the second PCR products of each sample used for library construction (Table S1).

### 2.8 Taxonomic assignment

The amplicon sequence variants were assigned to known taxa using the query‐centric auto‐k‐nearest‐neighbor (QCauto) method implemented in Claident (Tanabe & Toju, 2013). The QCauto method employs an iterative BLAST search for the query sequence to identify a borderline sequence, which can be regarded as the optimal threshold for delimiting candidate species when intra‐ and interspecies variation in the resultant taxa are considered. The query sequence is subsequently assigned to the lowest common ancestor (LCA), which is the lowest taxonomic level shared by the candidate species. Instead of identifying a query as a single species, QCauto provides a conservative assignment result. Owing to this algorithm, QCauto does not require a minimum similarity threshold to distinguish taxa (Tanabe & Toju, 2013). As a result, QCauto may be robust against splitting and lumping errors, which are unavoidable in ordinal similarity‐based clustering or discrimination methods with a single threshold value (Bonin et al., 2022). Furthermore, among the various assignment algorithms, QCauto returns the most reliable results when the completeness of the reference sequence database of all potentially observable species is low (Tanabe & Toju, 2013), as expected in the open ocean.

The homology search in QCauto was performed against the built‐in database of Claident comprising animal mitochondrial DNA to limit the candidates and reduce the computational time. The output for each ASV consisted of the resultant LCA and the GenBank accession number of neighborhood sequences upon which the assignment was based. The amplicon sequence variants assigned to taxa outside our target taxonomic groups, Actinopterygii and Elasmobranchii, were omitted from the following analysis.

The assignment results were modified based on the knowledge of the fish fauna around the study site. It was expected that QCauto would return an unnecessarily higher taxonomic level when referring to a database containing sequences of fish not distributed in the study area. Therefore, referring to a subset of species possibly present at the study site would return a more reasonable LCA.

In the modification, species not reported in the study area were eliminated from the original candidate species, and the LCA was revised based on a new set of candidates. First, the accession numbers returned by QCauto were searched against the NCBI database via Batch Entrez (https://www.ncbi.nlm.nih.gov/sites/batchentrez) to retrieve a list of candidate species. Next, the distribution area of these candidate species was retrieved from FishBase (Froese & Pauly, 2022) using the distribution function in rfishbase v.4.0.0 (Boettiger et al., 2012). The species reported in the FAO major fishing area corresponding to the study sites (18: Arctic Ocean for St. 10 and St. 35; 61: Pacific Northwest for P03 and K2) as endemic or native to the area were chosen as new candidates. Because these areas closely match the marine biogeographic realms (Costello et al., 2017), they provide a reliable reference for fish fauna. This information was supplemented by the latest complete species list of Japanese fishes (Motomura, 2020) for P03 and K2 and a species list of Arctic marine fishes (Sirenko et al., 2022) for St. 10 and St. 35. The report by Mecklenburg et al. (2018) was also used to filter out species that were only present in the Atlantic Arctic. For consistency, all species names were matched to valid names on FishBase using the validate_names function in rfishbase. When a valid name was not found, the presence of a species in the study area was checked using the GBIF database (https://www.gbif.org/).

After filtering candidate species, ASVs with identical sets of candidate species were consolidated to construct operational taxonomic units (OTUs). To take advantage of QCauto, which does not require a prerequisite similarity threshold for species identification (Tanabe & Toju, 2013), the identity of a set of candidates was adopted as a criterion for creating OTUs rather than clustering based on sequence similarity. The revised LCA was determined for each OTU based on a new set of candidate species using the lowest_common function in taxize v.0.9.99 (Chamberlain & Szocs, 2013) against the NCBI taxonomy database. For OTUs annotated above the genus level, the species name was left as “sp.” although it was impossible to determine whether the ASVs within such OTUs were derived from single or multiple species. When multiple OTUs shared the same LCA name above the genus level, they were differentiated by adding sequential numbers. The representative sequence of each OTU was determined as an ASV that had the most abundant read count in an OTU.

Any OTUs with suspicious assignments that returned an unnecessarily higher taxonomic level (higher than order) were corrected for errors based on the results of an online BLAST search (Appendix S1). The OTUs assigned to Exocoetidae sp. 1–8 were left separate to allow for the possibility that each OTU contained DNA derived from a distinct species, even though these OTUs partially shared candidate species (Appendix S1).

To confirm the genetic entities of the OTUs, the genetic similarity within each OTU (lowest similarity between representative sequence and its members in an OTU) and among OTUs (similarities among representative sequences) were computed using calc_distmx command in usearch (v11.0.667) and Pairwise Alignment function of JALview (version 2.11.2.5), respectively.

### 2.9 Statistical analysis

Species accumulation curves with respect to the number of corrected reads were constructed for each sample using the iNEXT function in iNEXT v.2.0.20 (Hsieh et al., 2016) to assess the sample completeness (Figure **S1**). Because all curves reached a plateau, it was assumed that the sequencing depth was sufficient to detect all of the species contained in a sample. Therefore, the results were not rarefied.

Asymptotic analysis was conducted using iNEXT based on the Hill number with order *q* = 0 to investigate the effect of increasing filtration volume on taxonomic richness obtained from eDNA metabarcoding. The Hill numbers are a set of diversity indices computed by a unified standardization function and monotonically change with the order *q*, which determines the weight given to the relative abundance of an OTU/ASV (Alberdi & Gilbert, 2019). When *q* = 0, the hill number corresponds to OTU richness. The Chao2 estimator (S_chao2_), which estimates true taxonomic richness based on observed data (Hsieh et al., 2016), was calculated using the following formula (Chao & Chiu, 2016).

$$S_{\text{chao}2}=\left\{\begin{array}{c} S_{\mathrm{obs}}+\left(\frac{T-1}{T}\right)\;\frac{Q_{1}^{2}}{2Q_{2}},\text{if}\;Q_{2}>0\\ S_{\mathrm{obs}}+\left(\frac{T-1}{T}\right)\frac{Q_{1}\left(Q_{1}–1\right)}{2},\text{if}\;Q_{2}=0 \end{array}\right.$$

where *S*_obs_ is the total number of detected taxa in a set of sampling replicates, *Q*_1_ and *Q*_2_ are the number of taxa occurring only once or only twice in a set of sampling replicates, and *T* is the number of filtration replicates. The rarefaction and extrapolation curves were defined as saturated when the accumulated number of detected taxa exceeded 95% of *S*_chao2_. Rare taxa that were detected in one or two filtration replicates were included in the analysis.

The compositional dissimilarity among replicates or between filters at each site was evaluated using turnover (*ß*_jtu_) and nestedness (*ß*_jne_) components of the Jaccard index (*ß*_jac_). The turnover component indicates the replacement of some species by others between samples while the nestedness component indicates the extent to which the taxonomic composition of a sample with a smaller number of species is a subset of a richer sample (Baselga, 2010, 2012). If the stochasticity of eDNA collection is low or a finer fraction of eDNA can improve species detection, nestedness is expected to be the dominant component. The dissimilarity indices were calculated using the beta.pair function in betapart 1.5.6 (Baselga, 2012) after converting the OTU‐by‐sample table into presence/absence data. The contribution of each component to total dissimilarity is expressed as the ratio of *ß*_jtu_ or *ß*_jne_ to *ß*_jac_. To evaluate the effect of filter pore size and increasing accumulated filtration volume on detected taxonomic composition, the dissimilarity indices were repeatedly calculated 100 times between filters or filtration volumes based on bootstrapped data, in which replicates of each filter were randomly pooled into identical volumes (3–9 L at K2 and St. 35 and 3–21 L at P03).

Differences in taxonomic composition among the filtration replicates for each site were visualized by nonmetric multidimensional scaling (NMDS) analysis based on the Jaccard dissimilarity using metaMDS function in vegan 2.6‐2 (Oksanen et al., 2022). Permutational multivariate analysis of variance (PERMANOVA; Anderson, 2017) was then performed using the adonis2 function in vegan 2.6‐2 (Oksanen et al., 2022) to examine the dissimilarity between filter types. In conjunction with PERMANOVA, the permutational dispersion test (PERMDISP; Anderson, 2006) was conducted using the betadisper function in vegan to assess the homogeneity of multivariate dispersion between the filter types.

Differences among the taxonomic richness detected per filtration in each set of sampling replicates were tested using Welch's *t*‐test when the assumption of normality was met after the Shapiro–Wilk test, or otherwise using Wilcoxon's rank‐sum test. Changes in compositional dissimilarity between filters associated with pooled filtration volume were tested using Spearman's rank correlation. The significance threshold was set at *p* < .05.

The relationship between the maximum filtration volume per filter and environmental factors expected to affect the clogging of filters (turbidity and chl‐*a* fluorescence) was assessed using multiple regression analysis using the lm function in R. Variables were log‐transformed to meet the normality assumption.

## 3 RESULTS

### 3.1 Sequencing statistics

After merging the paired‐end reads, a total of 4,062,982 reads for the replicated samples and 124,488 reads for the negative controls were obtained (Table S1). After quality filtering, denoising, and removal of the chimera sequences, 3,704,214 reads comprising 647 ASVs remained in the field sample. The negative control contained 3706 reads from 198 ASVs. Removing potential contaminants from the replicated samples following proportional correction of the read count resulted in 5,806,136 corrected reads from 479 ASVs. Of these, 427 ASVs were assigned to the taxa belonging to Actinopterygii (Table S2). No ASV was assigned to elasmobranchs. Fifty‐two ASVs assigned to mammals (*Homo sapiens, Sus scrofa, Odobenus rosmarus*, and cetaceans) were omitted from the results.

The ASVs assigned to Actinopterygii were consolidated into 93 OTUs based on composites of candidate species (Table S2). Of these, 47 OTUs consisted of multiple ASVs. Genetic similarity within and among OTUs was 96.5%–100% (99.4% in median) and 58.4%–99.4% (76.0% in median), respectively. Seventy‐four of these OTUs exhibited clear gaps between similarities of within and among OTUs. While, genetic similarities within and among OTUs overlapped in 13 pairs, comprising 19 OTUs, including five assigned to Exocoetidae, which had extremely low interspecific variation.

Few taxa predominated in the sequencing reads detected from each filtration replicate for both filter types. *Ammodytes hexapterus* dominated the detected reads at the Arctic sites, accounting for 96.9%–98.2% at St. 35 and 65.1%–91.2% at St. 10. (Figure S2). In the majority of filtration replicates at K2 and P03, only the top 2–4 taxa comprised over 80% of the total read counts. The taxa that accounted for over 30% of the total read counts in any filtration replicates were *Symbolophorus californiensis, Sardinops sagax*., Myctophidae sp.1, and *Engraulis japonicus* at K2, and *Gigantactis* sp., *Diplophos* sp.1, *Epinephelus areolatus*, and Phosichthyidae sp.1 at P03.

### 3.2 Comparison of taxonomic richness between filters and within replicates

**FIGURE 2:**
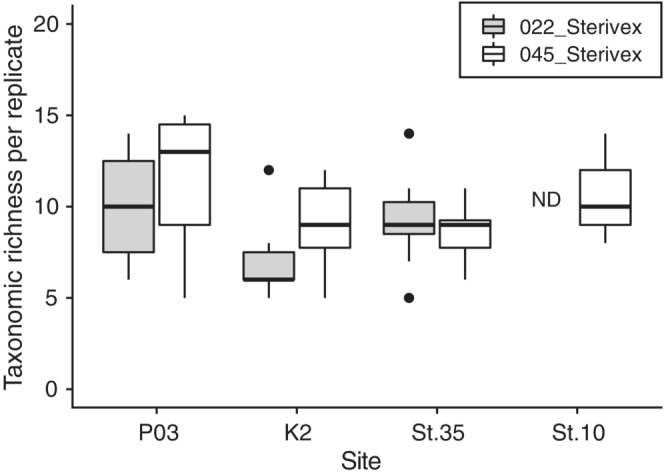
Comparison of taxonomic richness per filtration replicate for each filter type (022_Sterivex: 0.22 μm Sterivex‐GV and 045_Sterivex: 0.45 μm Sterivex‐HV) at the four study sites (P03, K2, St.35, and St.10). The bold lines in the boxes indicate medians, and the hinges of the boxes indicate the interquartile range (IQR; the first and third quartiles). Whiskers extend to the largest or smallest values no further than 1.5 × IQR from the hinge. Black dots represent outliers with values >1.5 × IQR. ND indicates no data.

The taxonomic richness detected per filtration replicate ranged from 5 to 15 taxa (Figure 2). There were no significant differences in taxonomic richness between filter types at any site (Welch's *t*‐test, *p* > .05). A total of 11–36 taxa were detected from seven to eight filtration replicates using 045_Sterivex across the four sites, whereas a total of 17–29 taxa were detected using 022_Sterivex across the three sites (Tables S3–S6). For both filters, taxonomic coverage represented by a single replicate was a small proportion of the total detected taxa at any site (36%–64% for 045_Sterivex and 36%–49% for 022_Sterivex). Taxa detected on a single replicate were prevalent in both filter types (9%–53% for 045_Sterivex and 32%–41% for 022_Sterivex).

**FIGURE 3:**
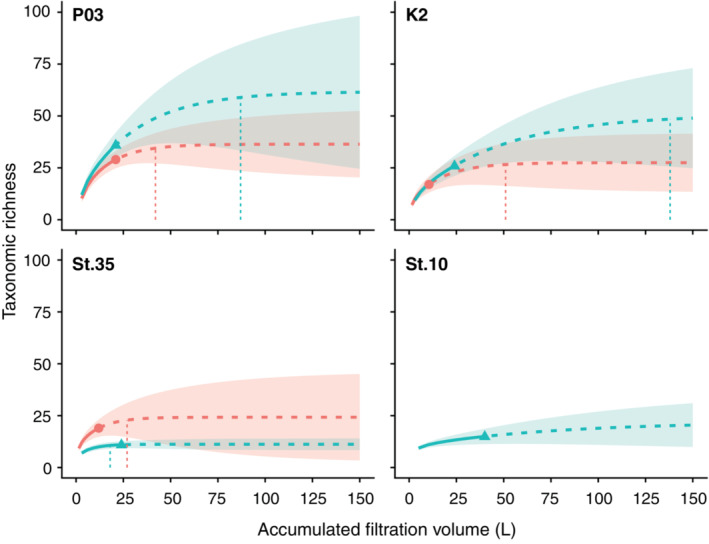
Rarefaction (solid line segment) and extrapolation (broken line segment) curves with 95% confidence intervals (shaded areas) for taxonomic richness obtained by eDNA metabarcoding with respect to accumulated filtration volume. The color of the line, area, and symbol corresponds to the filter types: 0.22 μm Sterivex‐GV (022_Sterivex) and 0.45 μm Sterivex‐HV (045_Sterivex) were denoted by red and blue, respectively. The symbols on the lines represent the observed value after aggregating all filtration replicates. Vertical dotted lines below the curves indicate the smallest accumulated filtration volume required to cover ≥95% of the Chao 2 estimator, except for St. 10, where the curve is saturated outside the range of the figure (175 L).

The estimated coverage per site, calculated as the ratio of the total number of detected taxa to *S*_chao2_, was 62%–80% for 022_Sterivex and 51%–98% for 045_Sterivex (Table 2). The accumulation curves indicated that the estimated species richness differed by filter even at the same site, with 045_Sterivex estimated to have higher richness At K2 and P03, while 022_Sterivex was estimated to have higher richness at St. 35 (Figure 3). However, the 95% confidence intervals for the extrapolation curves overlapped between the filters at all sites.

**TABLE 2:** Summary of the observed and estimated taxonomic richness of fish based on eDNA metabarcoding results. 022_Srteivex and 045_Sterivex denote 0.22 μm Sterivex‐GV and 0.45 μm Sterivex‐HV, respectively. The Chao2 estimator (*S*_chao2_) was indicated as an estimated value and 95% confidence interval (in the brackets). Coverage is the ratio of the number of detected taxa to *S*_chao2_.

Station	Filter types	Number of detected taxa	*S*_chao2_	Coverage (%)	Accumulation volume covering ≥95% of *S*_chao2_ (L)	Number of filtration replicates covering ≥95% of *S*_chao2_
St10	045_Sterivex	15	22 [16, 72]	68	175	35
St35	022_Sterivex	19	24 [20, 49]	78	27	18
	045_Sterivex	11	11 [11, 15]	98	18	6
K2	022_Sterivex	17	28 [19, 76]	62	51	34
	045_Sterivex	26	51 [32, 127]	51	138	46
P03	022_Sterivex	29	36 [31, 58]	80	42	14
	045_Sterivex	36	62 [44, 118]	58	87	29

### 3.3 Comparison of taxonomic composition between filters and within replicates

**FIGURE 4:**
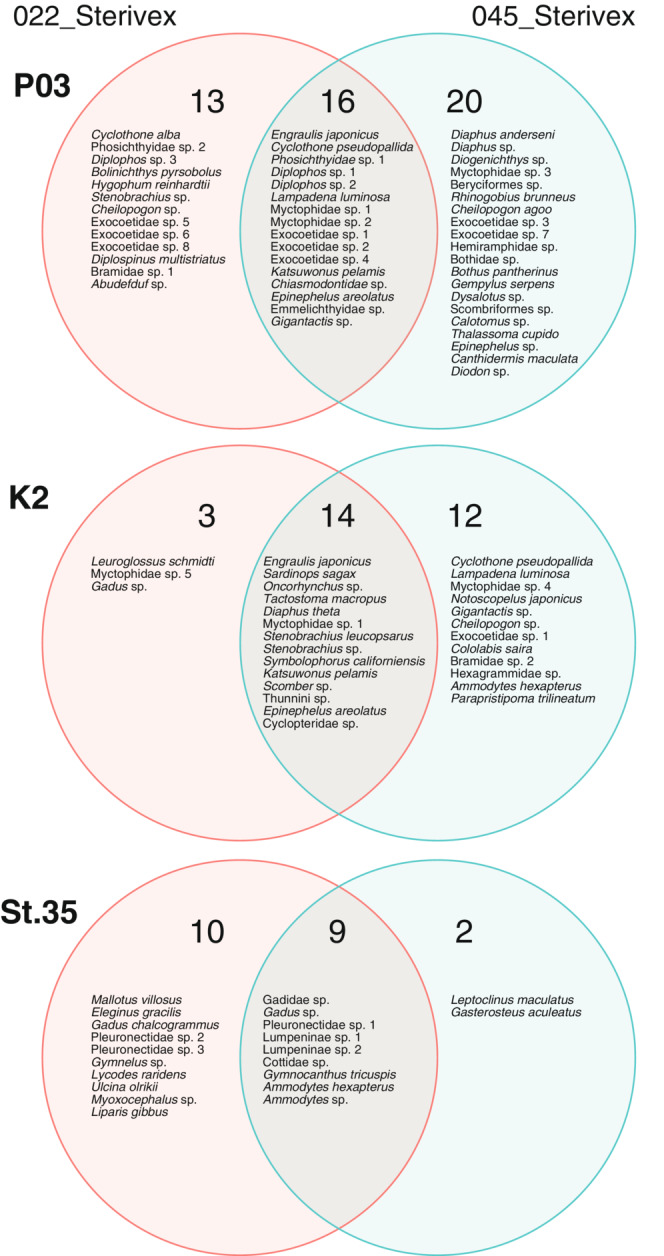
Venn diagram showing overlap between taxonomic compositions obtained from eDNA metabarcoding of samples collected using two different types of filters (022_Sterivex: 0.22 μm Sterivex‐GV and 045_Sterivex: 0.45 μm Sterivex‐HV). The sizes of the circles were not proportional to the number of taxa detected.

Remarkable differences in taxonomic composition between the two filter types were found after scrutinizing the taxonomic components (Figure 4; Tables S3–S5). At P03, the taxonomic composition obtained from 022_Sterivex and 045_Sterivex partially overlapped (33%), with each filter producing a distinct composition. The number of uniquely detected taxa was 13 in 022_Sterivex (27%) and 20 in 045_Sterivex (41%). However, at K2, the detected taxa from 045_Sterivex comprised 90% of all detected taxa. Only three taxa were detected uniquely by 022_Sterivex. Contrary to K2, 022_Sterivex produced a more comprehensive result at St. 35, accounting for 90% of the whole detected taxa. Only two unique taxa were detected in 045_Sterivex. Compared with the taxa commonly detected across filters, the taxa that appeared uniquely in each filter were detected less frequently, with the majority of them only appearing once (73% at P03, 93% at K2, 50% at St. 35) in filtration replicates (Tables S3–S5).

**FIGURE 5:**
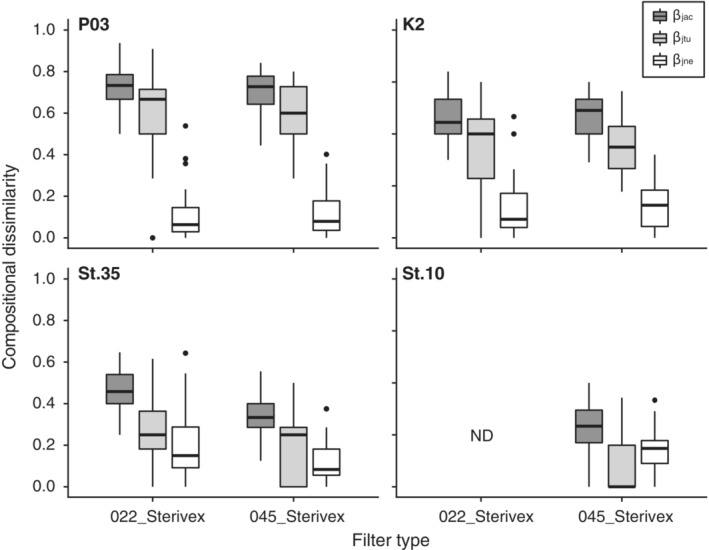
Compositional dissimilarity between filtration replicates across the four study sites (P03, K2, St. 35, St. 10) and two different types of filters (022_Sterivex: 0.22 μm Sterivex‐GV and 045_Sterivex: 0.45 μm Sterivex‐HV). The Jaccard index (*β*_jac_) was partitioned into turnover (*β*_jtu_) and nestedness components (*β*_jne_). The bold lines in the boxes indicate medians, and the hinges of the boxes indicate the interquartile range (IQR; the first and third quartiles). Whiskers extend to the largest or smallest values no further than 1.5 × IQR from the hinge. Black dots represent outliers with values >1.5 × IQR. ND indicates no data.

Consequently, the Jaccard indices between the filter types were relatively high or moderate even in the same site (*ß*_jac_: mean ± SD = 0.76 ± 0.11 at P03, 0.57 ± 0.12 at K2, and 0.44 ± 0.12 at St. 35). Comparisons among filtration replicates within the same filter type yielded a similar degree of Jaccard indices as between the filter types (Figure 5). Partitioning the Jaccard index indicated that turnover was the main component of compositional dissimilarity among filtration replicates for both filter types in all study sites, except at St. 10. At P03 and K2, the majority of the compositional dissimilarity was due to turnover (*ß*_jtu_ to *ß*_jac_: mean = 80.3% for 022_Sterivex and 82.5% for 045_Sterivex at P03, 72.5% for 022_Sterivex and 73.1% for 045_Sterivex at K2), whereas the contribution of nestedness (*ß*_jne_) was very limited. At St. 35, the turnover exceeded the nestedness, but its dominance was relatively moderate (55% for 022_Sterivex, 60% for 045_Sterivex). Contrary, Nestedness was the dominant component in compositional dissimilarity at St. 10 (69%), although only 045_Sterivex was tested.

**FIGURE 6:**
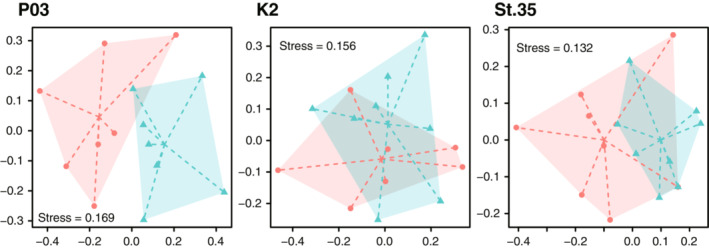
Nonmetric multidimensional scaling (nMDS) ordinations based on the Jaccard dissimilarity showing differences in taxonomic composition among filtration replicates. Red circles and blue triangles indicate 0.22 μm Sterivex‐GV (022_Sterivex) and 0.45 mm Sterivex (045_Sterivex), respectively. The hulls represent the dispersion of the data, whereas the lines represent the dispersion around the centroids.

Nonmetric multidimensional scaling ordinations based on the Jaccard indices showed discernible differences in taxonomic composition between 022_Sterivex and 045_Sterivex at P03, whereas its dispersion was highly overlapping between the filter types at K2 and St. 35 (Figure 6). PERMANOVA revealed statistically significant differences in taxonomic composition between filter types at P03 and St. 35 (*p* < .05), but not at K2 (Table S7). Additional PERMDISP analysis indicated that the dispersions of the compositions were equivalent between filter types at all sites (*p* > .1, Table S8), suggesting that the significant differences in the PERMANOVA results for P03 and St. 35 were due to the differences in the position of the centroids.

**FIGURE 7:**
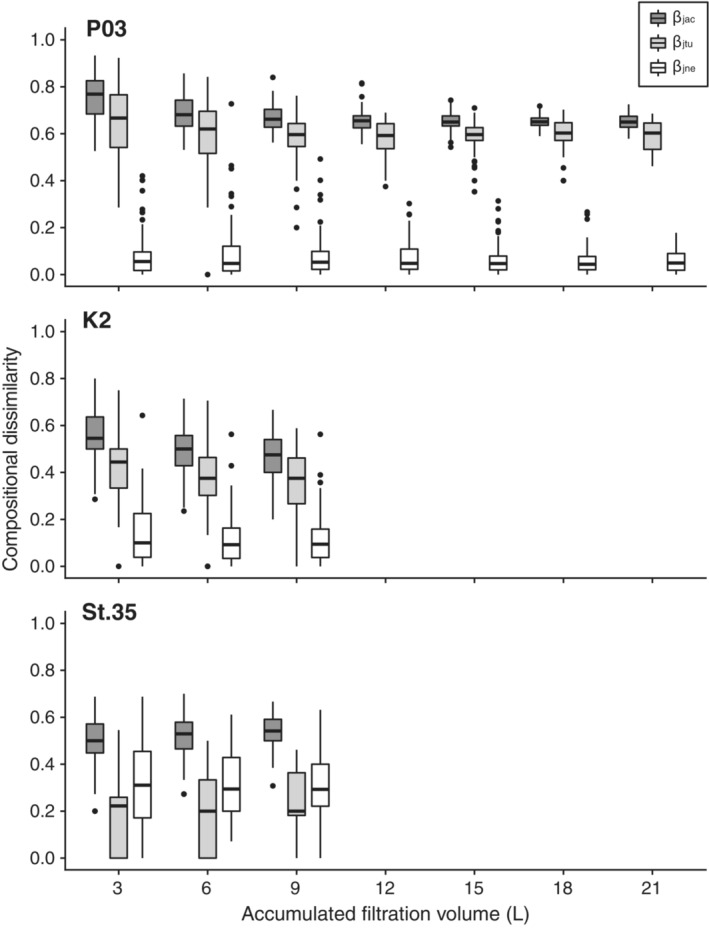
Changes in compositional dissimilarity between filters (022_Sterivex: 0.22 μm Sterivex‐GV and 045_Sterivex: 0.45 μm Sterivex‐HV) associating with accumulated filtration volume. The Jaccard index (*β*_jac_) was partitioned into turnover (*β*_jtu_) and nestedness components (*β*_jne_). The bold lines in the boxes ndicate medians, and the hinges of the boxes indicate the interquartile range (IQR; the first and third quartiles). Whiskers extend to the largest or smallest values no further than 1.5 × IQR from the hinge. Black dots represent outliers with values >1.5 × IQR.

Increasing the accumulated filtration volume by pooling the results of multiple filtration replicates had only a limited effect on reducing the compositional dissimilarity between filter types (Figure 7). Despite a significant correlation (Spearman's rank correlation, *p* < .05) between accumulated filtration volume and the Jaccard index, increasing the volume had only a minor effect on the Jaccard index between the filters at P03 and K2. The Turnover component (*ß*_jtu_) always dominated the compositional dissimilarity between two types of filters (*ß*_jtu_ to *ß*_jac_: 86.6%–91.5% at P03, 75.1%–76.6% at K2), with a minor contribution from nestedness (*ß*_jne_). In contrast, the Jaccard indices between filters at St. 35 increased significantly with increasing accumulated filtration volume (Spearman's rank correlation, *p* < .05). In this case, compositional dissimilarity was mostly due to nestedness (*ß*_jne_ = 59.5%–65.8%) with a lesser contribution from turnover (*ß*_jtu_ = 34.2%–40.5%).

### 3.4 Evaluating sampling effort

The rarefaction and extrapolation curves predicted that a substantial filtration volume was required to saturate taxonomic richness (approximately 42–51 L for 022_Sterivex and 87–138 L for 045_Sterivex), except at St. 35, where approximately 20–30 L of filtration was expected to be required to approach an asymptote (Figure 3). The number of filtration replicates required to saturate the extrapolation curves was also remarkably higher for 045_Sterivex, at 29–46 replicates compared to 14–34 replicates for 022_Sterivex (Figure S3). One exception is St. 35, where only six replicates were required to saturate the extrapolation curve for 045_Sterivex.

A single 045_Sterivex filter filtered approximately 5 L of seawater in the northwestern Pacific and the Bering Sea and approximately 9 L in the Arctic Ocean until it was clogged (Figure S4). A single filtration process took approximately 80–100 min. The average filtration rates were approximately 59 mL/min in the northwestern Pacific, 67 mL/min in the Bering Sea, and 87 mL/min in the Arctic Ocean. Significant negative effects of the turbidity and chl‐*a* concentration on the total filtration volume were found (multiple regression analysis, turbidity: *t* = −3.684, *p* < .001, chl‐*a* fluorescence: *t* = −7.222, *p* < .001, adjusted *R* ^2^ = .881).

## 4 DISCUSSION

This study demonstrated that the stochasticity of eDNA collection had a significant impact on the metabarcoding‐based detection of fish eDNA, while the pore size of the filter appeared to have little or no effect. Additionally, the species accumulation curves suggested that the number of detected taxa did not reach a plateau in most cases, indicating that the number of sample replicates collected per site (7 or 8 samples, corresponding to 10.5–40 L of filtration in total) was insufficient to fully assess the species diversity in the open ocean. The presence of rare taxa across all sites and filters implied that fish eDNA of many species is contained in the seawater at low concentrations, so capturing fish eDNA is dependent on random chance. Even when a standardized sampling protocol is utilized at a single site, stochasticity will lead to inconsistency in snapshots of fish species composition. This is undesirable for tracking the spatiotemporal pattern of fish diversity. Therefore, to optimize eDNA sampling strategies for practical use in the open ocean, it is necessary to disentangle how sampling effort and filter pore size affect the collection of fish eDNA.

### 4.1 Effect of sampling effort on fish eDNA detection

The rarefaction and extrapolation curves with respect to the filtration volume and *S*_chao2_ calculated based on the eDNA metabarcoding results indicated that several sampling replicates or filtering a large amount of seawater (tens or hundreds of liters of seawater) were required to completely detect fish species that possibly occur around the study sites. This result was consistent with those of previous studies using eDNA metabarcoding in inland water and coastal areas (Bessey et al., 2020; Cantera et al., 2019; Stauffer et al., 2021). Consequently, employing a typical sampling effort (filtering 0.5–2 L without sampling replicates) would likely be insufficient when performing sampling in the open ocean and will lead to an underestimation of fish diversity.

The need for several sampling replicates or filtration of a mass amount of water to maximize species detection is probably owing to the low concentration of fish eDNA contained in open ocean seawater, as expected from the results reported by Stat et al. (2017). Because the degradation, transportation, and dilution of eDNA are important for eDNA detection in the ocean, the detection probability of fish eDNA is expected to rapidly decrease with increasing distance from the source organism and time from shedding (Collins et al., 2018; Hansen et al., 2018; Thomsen et al., 2012). In particular, dilution is thought to have a critical effect that lowers fish eDNA detectability in the open ocean, coupled with a low water volume‐to‐biomass ratio of fish. Although the eDNA half‐life is significantly longer in offshore water than in inshore water and the decay rate varies depending on the environment and source organisms (Collins et al., 2018), it is possible that the eDNA concentration decreases to below the lowest detectable concentration within a few hours or days.

This hypothesis is reasonably supported by the results of the present study. The compositional dissimilarity was dominated by the turnover component, indicating that species replacement frequently occurred between samples, even when filtering seawater concurrently collected from a single site. In addition, the high dissimilarity between filters was not mitigated and turnover consistently dominated with increasing accumulated filtration volume at P03 and K2. This pattern of dissimilarity is likely due to the prevalence of rare taxa. Although metabarcoding does not quantify the eDNA contained in the extracts, the relative abundance of the resultant reads suggests that the eDNA shed from abundant species has high detectability (Kelly et al., 2014). Operational taxonomic units frequently found across replicates were assigned to fish that were abundant in the pelagic or mesopelagic ocean (*Ammodytes hexapterus*, *Sardinops sagax*, and various lanternfishes). These fishes might have overwhelmed the sequencing reads because of their abundance as eDNA sources. In contrast, rare taxa tended to occupy a small fraction of the detected reads. These results imply that eDNA from most fish species was present in the open ocean, with a density of a few particles per tens of liters of seawater as a detectable state.

Because the estimated sampling effort for the comprehensive detection of fish eDNA is excessively high, finding a balance between the risk of underestimating fish community composition and allowable sampling effort is essential to identify the optimal protocol for eDNA collection. Combining the results of the asymptotic analysis and the estimation of the feasible sampling effort in this study provides valuable information for determining the optimal sampling effort for eDNA collection. A single 045_Sterivex could filter 5–9 L in approximately 2 h in our study area. Usually, 3–6 Sterivex filters can be handled simultaneously using a peristaltic pump for pressure filtration or a manifold for vacuum filtration. This means that the realistic sampling effort per site would be 15–50 L of water filtered every 2 h with 3–6 filtration replicates. Based on the accumulation curves in this study, it is expected that this accumulated filtration volume would provide 49%–98% coverage at P03, 41%–95% coverage at K2, and 84%–100% at St. 35. Considering the cost for collection, transportation, and filtration on board, this effort is quite realistic. However, the trade‐off between sensitivity to detect rare taxa and sampling effort was not fully explored here. Appropriate sampling strategies should be determined depending on the study's purpose.

One caveat of the asymptotic analysis used in this study is that the high proportion of rare taxa may have confounded the estimation of species richness. An asymptote in the accumulation curve provides a reasonable estimation of the true diversity of the subjected community and the sampling effort required to completely assess the community composition. However, asymptote estimators, such as the Chao2 and Jackknife estimators, are sensitive to sample completeness, as measured by sample coverage (Roswell et al., 2021). The high proportion of rare taxa likely inflated the confidence interval of *S*_chao2_ at all sites. Therefore, it is possible that the coverage of the estimated true fish diversity in this study was uncertain. Another source of uncertainty in this study was the fact that the filtration volume per filter was inconsistent among the filters. This study assumed that if the total filtration volume was equal, integrating the results from sample replicates would produce a comparable taxonomic composition regardless of the number of replicates created. However, increasing the number of filtration replicates entails an increase in the effort in subsequent analysis, such as the number of PCR replicates and sequencing depth, which improves the probability of eDNA detection (Doi et al., 2019; Ficetola et al., 2015). An optimal balance of the efforts in eDNA sampling and downstream molecular work needs to be determined in the future.

The optimal sampling effort discussed above may partially detect the true diversity at the study site but will produce estimations comparable with those obtained by the conventional method. Another approach to evaluate the optimal sampling effort according to the detection power of eDNA metabarcoding is to compare species richness and composition between eDNA surveys and investigations using conventional methods (Thomsen et al., 2016). Although the paucity of inventory data on fish fauna in the vicinity of our study sites precludes the evaluation of the detection power of eDNA, the results obtained from the Chukchi Sea are an excellent example of demonstrating the usefulness of eDNA in describing local fish fauna. It has been suggested that the Chukchi Sea possesses the most diverse fish fauna in the Arctic region, with at least 110 fish species (including freshwater fish) reported (Datsky, 2015). Previous trawling surveys have recorded 24 fish species from the near‐shore Alaskan coast and 24 species from the northern Bering to the Chukchi Sea shelf (Lin et al., 2014; Thedinga et al., 2013). The taxa detected in this study highly overlapped with previous results and the total number of detected taxa (11–19 taxa) was comparable with the total number of species reported in the above study. Although the species composition from eDNA could not be directly compared with previous results at the species level because the eDNA marker does not have enough power to discriminate all known species (Collins et al., 2019), the results obtained from eDNA metabarcoding seemed to represent the local fish fauna.

### 4.2 Effect of pore sizes on fish eDNA detection

The current study suggested that the compositional difference between filter types found at subtropical (P03) and subarctic (K2) sites could not be attributed to differences in the efficiency of eDNA collection but rather to the stochastic nature of eDNA collection. At P03, a venn diagram clearly illustrated that taxonomic composition overlapped little between filter types, with each filter containing a high proportion of uniquely detected taxa. The results of NMDS and PERMANOVA indicated that the taxonomic composition differed significantly between filter types. The Jaccard index between filtration replicates was comparable with that between filter types. Compositional dissimilarity was dominated by the turnover component for both replicates within a filter type and between filter types. Even though the taxonomic composition was not statistically distinct, K2 also exhibited a similar trend of dissimilarity. These results contradict an expectation based on the assumption that 022_Sterivex collects eDNA more efficiently than 045_Sterivex, in which 022_Sterivex detected more taxa than 045_Sterivex and the nestedness component dominated the dissimilarity between filters. Furthermore, increasing the sampling effort only slightly reduced the dissimilarity between filter types. These results indicated that the selection of filters had a negligible effect on the detection of fish eDNA, most likely due to the stochastic nature of eDNA collection and the low density of fish eDNA.

Meanwhile, the results from the Chukchi Sea shelf (St. 35) suggested that 022_Sterivex was capable of capturing eDNA from a broader range of taxa than 045_Sterivex, though the effect of stochastic collection on eDNA detection cannot be ruled out. In contrast to P03 and K2, the contributions of nestedness and turnover to the total compositional dissimilarity between filters at St. 35 were comparable. This pattern, as well as the overlap in taxonomic composition between filters, indicated that the fish community detected by 045_Sterivex was a strict subset of the fish community detected by 022_Sterivex. Since many species were detected only once or twice, this result could be attributed to the low density of fish eDNA. However, the high contribution of nestedness likely supports an alternative hypothesis in which the 022_Sterivex could effectively capture eDNA comprising <0.45 μm fraction of particles and consequently improve the detection probability of fish eDNA.

These findings suggest that the effect of filter selection on the collection of fish eDNA varies depending on the study area. The most plausible explanation for this variability is that eDNA undergoes a site‐specific degradation process and has a different PSD at each study site due to the unique environmental conditions. Recent studies have suggested that the >0.2 μm size fraction contains the most detectable eDNA, with the 1–10 μm size fraction containing the largest proportion (Barnes et al., 2021; Cooper et al., 2022; Jo et al., 2019; Zhao et al., 2021). Because the size range of a vertebrate mitochondrion is 0.2–8 μm, a nominal fraction below this size is thought to contain degraded DNA, such as extracellular and extra‐organellar DNA free in solution or its aggregates (Turner et al., 2014). Considering the PSD shift toward a smaller size fraction with degradation (Jo et al., 2019), the positive effect of using 022_Sterivex observed in St. 35 may be a result of the accumulation of degraded eDNA in <0.45 μm size fraction. Because the Water temperature is a significant environmental factor that influences the decay rate of eDNA (Jo & Minamoto, 2021), it is probable that the extremely low water temperature in the Arctic region may suppress eDNA degradation and enhance its accumulation in the <0.45 μm size fraction. Furthermore, the Arctic fish fauna is less diverse than the subtropical fish fauna, with distinct taxonomic compositions dominated by eelpouts (Zoarcidae), sculpins (Cottidae), and snailfishes (Liparidae) (Mecklenburg et al., 2018). Differences in physiology and behavior between Arctic and subtropical fish may also contribute to eDNA persistence and PSD.

Although the effect of filter pore size on eDNA detection was expected in the Arctic, the prevalence of rare taxa in each filtration experiment in this study suggested that the sparse distribution of fish eDNA may regulate detection patterns in the open ocean. According to this finding, it could be expected that the collection of such sparse fish eDNA was highly stochastic and would result in heterogeneous detection among filtration replicates and between filters, even when seawater collected at the same site was filtered simultaneously. Especially in the subtropical region, which possesses highly diverse fish fauna and possibly a higher decay rate of eDNA compared with the polar region, it can be speculated that fish eDNA dispersed heterogeneously in the seawater with low concentrations in the degraded state. Therefore, the detection of fish eDNA did not improve even when using a fine filter.

### 4.3 Possible adverse effects of excessive sampling effort

Although this study showed that increasing the filtration volume improved the detection performance of fish eDNA, an adverse effect caused by excessive effort should be addressed to optimize eDNA collection. Previous studies have demonstrated that using a capsule filter with a high capacity allows the filtration of a mass volume of water at a high filtration rate (approximately 30 L per filter and over 1 L/min; Civade et al., 2016; Cantera et al., 2019; Goutte et al., 2020; Prié et al., 2021; Valentini et al., 2016). However, although an increase in filtration volume can result in a high eDNA yield (Hunter et al., 2019), there is a risk of decreasing extraction efficiency due to sample overload when following the column extraction procedure (Wong et al., 2020). The extraction process from the capsule filter usually involves the resuspension and precipitation of particles (Cantera et al., 2019), unlike the standard extraction protocol for Sterivex filters (Wong et al., 2020). This protocol is more complicated and laborious than extraction from Sterivex and could potentially decrease the recovery yield of DNA due to sample loss during the discarding of the supernatant. Another concern is the accumulation of various PCR inhibitors, such as humic acid and nontarget DNA, with the mass filtration volume. Therefore, although using a large‐capacity filter is a possible strategy to improve fish eDNA detection in the open ocean, its performance should be clarified further in future experimental studies.

Detecting nonlocal and nontemporary eDNA originating from spatially and temporally distant sources from a study site would cause false‐positive errors (Hansen et al., 2018). Unlike enclosed environments, such as lakes, rivers, or bays, evaluating the completeness and validity of eDNA results is more difficult in the open ocean because the distribution extent of each species cannot be identified. Therefore, even if increasing the sampling effort and use of fine filters can improve the collection of sparsely distributed and degraded rare eDNA, there is an inherent risk of obtaining an overview that misleads the estimation of the distributional range of fish. A field experiment conducted in a bay with a weak current showed that eDNA shed from a marine fish (*Pseudocaranx dentex*) can be detected within a range of <100 m (Murakami et al., 2019). Considering this narrow detectable range and the expected spatial resolution in an expedition, the increasing risk of false‐positive detection could be negligible in an eDNA survey conducted in the open ocean. However, fish eDNA in the ocean show a variety of decay rates, with half‐lives of approximately 7 h to 3 day, and degrade more slowly in offshore water than in inshore water (Collins et al., 2018). Cowart et al. (2018), who investigated eDNA in the West Antarctic Peninsula, estimated that detectable amounts of eDNA of ocellated icefish, *Chionodraco rastrospinosus*, might be dispersed as far as 648 km from its source in 25 days, assuming a current velocity and slow decay rate under subzero water temperatures. Understanding the degradation and dispersal processes in the open ocean would contribute to adequately evaluating the performance of eDNA detection and optimizing sampling methods to avoid possible adverse effects.

### 4.4 Recommendations

This study emphasizes that stochastic collection of fish eDNA, due to its sparse distribution, can have a profound effect on the results of the eDNA metabarcoding survey in the open ocean and possibly provide a biased overview of fish diversity there. Increasing the sampling effort (i.e., total filtration volume or filtration replicate) is thought to be more beneficial than filter selection for extensive and comprehensive detection of fish eDNA. It is also expected that analyzing spatial and temporal replicates of eDNA samples to account for the heterogeneity of eDNA distribution will result in a more integrative and consistent estimation of fish community composition, though this possibility was not explored in this study. However, more extensive sampling requires more time and manpower, which could lead to higher analytical costs (Fukaya et al., 2022). Therefore, we suggest that the sampling strategy of eDNA for investigating fish diversity and distribution in the open ocean should be optimized for practical use by maximizing the robustness and reproducibility of metabarcoding results with allowable sampling effort, rather than aiming for complete detection of local fish fauna.

The important implication of this study is the usefulness of asymptotic analysis with replicated sampling in a pilot study to construct an optimal sampling strategy of eDNA for ecological and monitoring purposes. Especially in the open ocean, where comparable data on fish fauna are often unavailable, the results from an asymptotic analysis could be crucial for evaluating the effectiveness of the eDNA approach. Therefore, for practical sampling, we recommend using multiple filtration replicates per site as a standard eDNA collection protocol. Integrating the results from multiple filtration replicates (at least triplicate or more) allows the construction of interpolation and extrapolation curves with respect to sampling effort, and species richness estimators, such as those analyzed in this study.

## 5 CONCLUSION

The open ocean is a mosaic of distinct water masses with characteristic hydrographic conditions and water chemistries. It is expected that this heterogeneity in the environment at various spatial and temporal scales will produce regional differences in eDNA persistence and degradation processes. For example, the Arctic shows a unique environment with low water temperature and seasonal or permanent sea ice cover and supports diverse fish species adapted to a low‐temperature environment (Eamer et al., 2013). Under such harsh conditions, it can be hypothesized that the ecology of eDNA (i.e., origin, state, transport, and fate within the environment; Barnes & Turner, 2016) differs in temperate and tropical oceans. As a result, the optimal sampling protocol will also vary across regions, which will be an obstacle in standardizing the sampling strategy and protocols across the open ocean. Therefore, accumulating basic knowledge of the ecology of eDNA in various environments in the open ocean is required to develop the practical use of eDNA. Using a statistical model, such as a multispecies site occupancy model that estimates occurrence/detection probabilities of species based on eDNA detection, will help to determine an efficient sampling strategy and optimal sampling effort (Doi et al., 2019; Fukaya et al., 2022). Further studies attempting to integrate field experiments and statistical approaches will contribute to establishing a basis for developing an optimal eDNA survey for the open ocean.

## AUTHOR CONTRIBUTIONS

**Tatsuya Kawakami:** Conceptualization (equal); data curation (lead); formal analysis (lead); investigation (lead); methodology (lead); visualization (lead); writing – original draft (lead); writing – review and editing (lead). **Aya Yamazaki:** Formal analysis (supporting); writing – review and editing (supporting). **Maki Asami:** Formal analysis (supporting); writing – review and editing (supporting). **Yuko Gotoh:** Formal analysis (supporting); writing – review and editing (supporting). **Hiroki Yamanaka:** Formal analysis (supporting); writing – review and editing (supporting). **Susumu Hyodo:** Writing – review and editing (supporting). **Hiromichi Ueno:** Conceptualization (equal); funding acquisition (equal); project administration (equal); resources (equal); supervision (equal); writing – review and editing (supporting). **Akihide Kasai:** Conceptualization (equal); funding acquisition (equal); project administration (equal); resources (equal); supervision (equal); writing – review and editing (supporting).

## BENEFIT‐SHARING STATEMENT

Benefits Generated: All sequence data used in this study have been deposited and publicly shared via the appropriate database described above and will benefit future studies relevant to the conservation and sustainable use of biological diversity.

## Supporting information


Appendix S1

## Data Availability

Raw sequence data files were deposited in the DNA Data Bank of Japan (DDBJ) Sequence Read Archive (DRA) as No. DRA014723.
