# Supplementary material for: Evaluating the sampling effort for the metabarcoding‐based detection of fish environmental DNA in the open ocean
Source: Ecol Evol. 2023 Mar 24;13(3):e9921. doi: 10.1002/ece3.9921 (PMC10037434; doi:10.1002/ece3.9921)
Supplement: Supplementary file 1 — Appendix S1 [file ECE3-13-e9921-s001.pdf]

Supporting information 1

Correction of suspicious taxonomic assignments

Suspicious assignments were inspected and corrected for errors. Two Cyprinoidei sp. were discarded because the presence of cyprinoids in the open ocean is doubtful, considering their restricted distribution in fresh or brackish water. For other OTUs assigned to taxa above the order level, an online BLAST search (https://blast.ncbi.nlm.nih.gov/Blast.cgi) was performed for each sequence against the nr/nt database (June 28, 2022). Two Euteleosteomorpha sp. showed a significant hit with *Symbolophorus californiensis*, with ≥98% similarity. Scombriformes sp.1 and Eupercaria sp. matched *Diplospinus multistriatus* and *Parapristipoma trilineatum*, respectively, and were distinguishable from the other species with <90% similarity. The annotations of these OTUs were modified according to the species name of the nearest sequence. Scombriformes sp.2 showed significant matches with multiple scombriform fishes with high similarities (≥97%). For Beryciformes sp., no closely matched sequence was found (<91%), but the species with the highest similarity was the beryciform species, *Scopeloberyx robustus*. Therefore, the annotations of these OTUs remained at the order level. *Sardinops* sp. was revised to *S. sagax* because it adopted *S. sagax* as a valid name, consolidating *S. sagax* and its pacific counterpart *S. melanostictus*.

Eight OTUs with four to nineteen candidate species from the genera *Cypselurus, Cheilopogon, Exocoetus*, and *Hirundichthys* were assigned to Exocoetidae sp. 1-8 (Table S2). Because these OTUs partially shared candidate species and showed high sequence similarity in some pairs (over 98.5%), there was a risk of artificially inflating the taxonomic diversity when each resultant OTU was regarded as a distinct species. However, considering that Exocoetidae lack sufficient interspecific variation in the 12S ribosomal RNA gene region of mitochondrial DNA to distinguish species, merging these OTUs would result in an underestimation of the true diversity of Exocoetidae. Given the discrepancies between genetically distinct OTUs and the known species recognized by traditional taxonomy, evaluating the true diversity of the Exocoetidae from their eDNA is extremely difficult.

In this study, the OTUs assigned to Exocoetidae sp. 1–8 were regarded as distinct species, prioritizing the possibility that they originated from different species. Exocoetidae are pelagic fishes with a wide range of distribution in the open ocean. Because 28 exocoetids have been reported in one of our study areas, the waters surrounding Japan (Motomura, 2020), eDNA from multiple exocoetids is expected to be detected sympatrically. Furthermore, the difficulty of species-level discrimination in Exocoetidae is most likely due to the lack of interspecific variation rather than a high degree of intraspecific variation. In this situation, taxonomic assignment based on sequence similarity is more likely to lead to merging errors than splitting errors. Therefore, this decision implies that the risk of splitting error due to insufficient interspecific variation was accepted rather than lowering the sensitivity to detect species due to merging error.

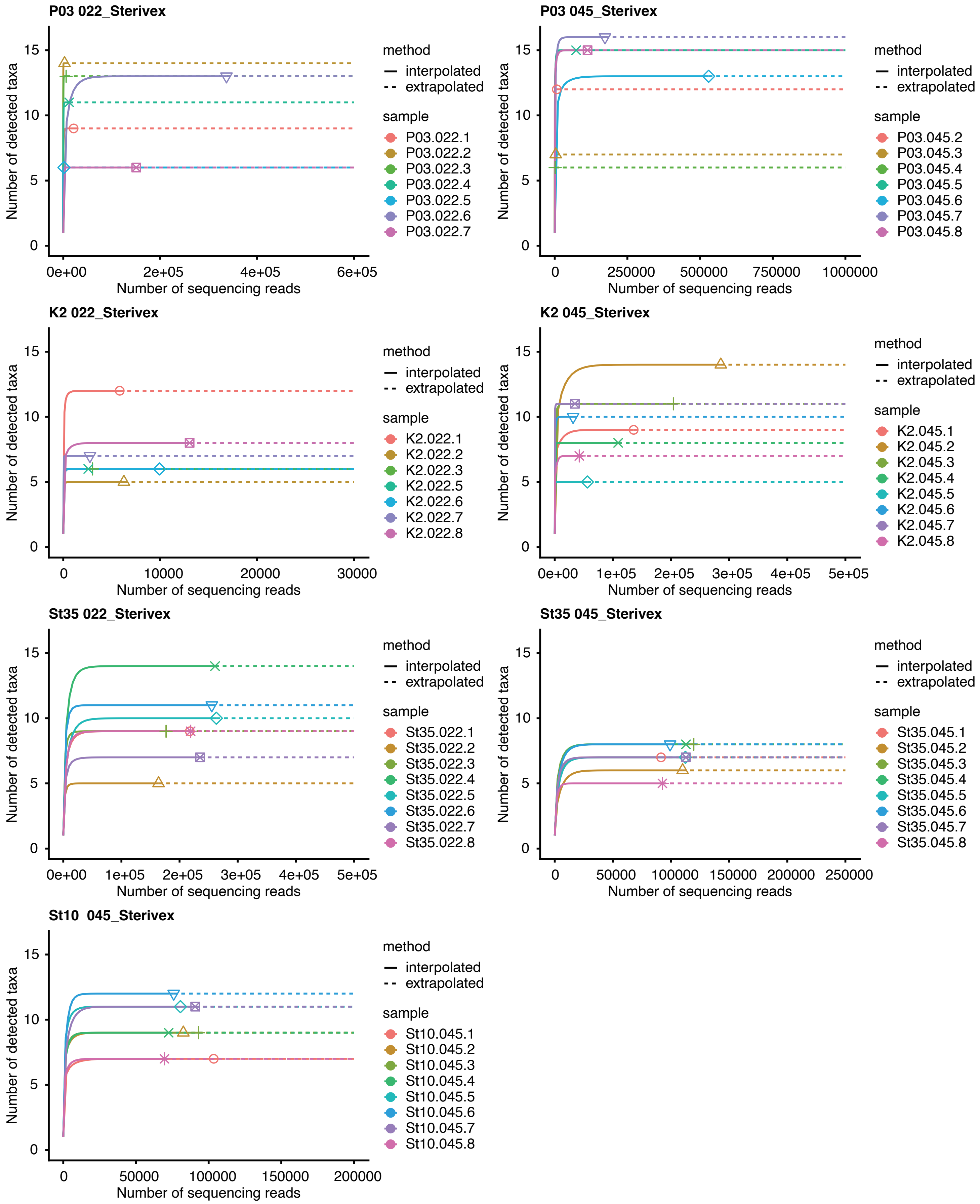

Figure S1 Accumulation curves of the number of detected taxa against the number of sequencing reads across the four study sites (P03, K2, St.35, and St.10) and two different types of filters (022_Sterivex: 0.22 μm Sterivex-GV and 045_Sterivex: 0.45 μm Sterivex-HV). Read counts were corrected proportionally to the volume of PCR product mixed in a sequencing sample. Because the data was obtained across three sequencing runs, this correction was individually applied to each run. The symbols indicate the observed value, and the solid and dotted lines indicate the rarefaction and extrapolation curves, respectively.

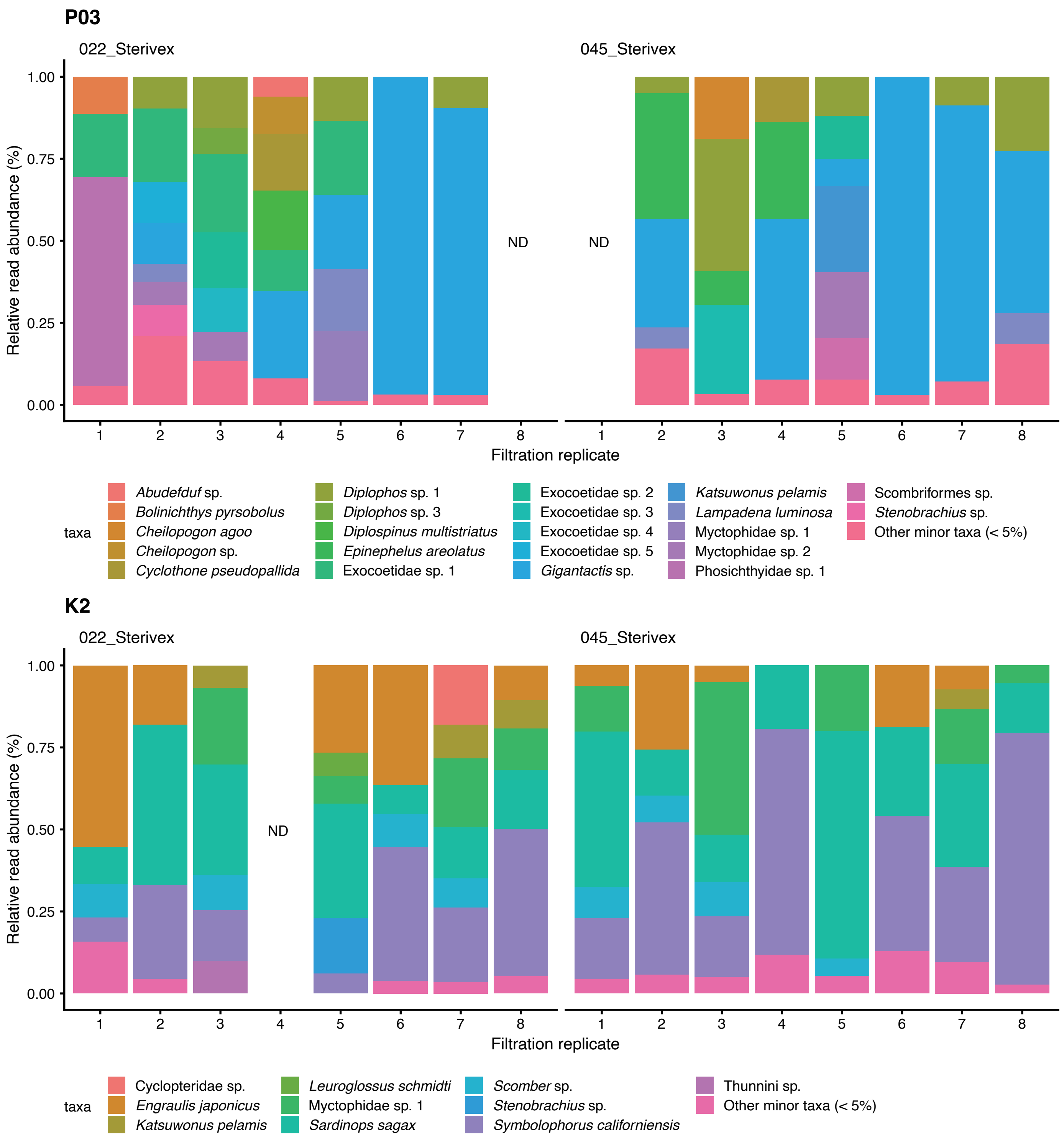

Figure S2 Relative read abundance of fish taxa obtained by eDNA metabarcoding from the sites in the subtropical (P03) and subarctic (K2) gyres of the northwestern Pacific Ocean, and the shelf (St. 35) and the slope (St. 10) of the Arctic Chukchi Sea. 022_Srteivex and 045_Sterivex denote 0.22 μm Sterivex-GV and 0.45 μm Sterivex-HV, respectively. The result from each filtration replicate was indicated separately within site. ND in panels indicates no data because of a lack of appropriately collected filtration replicates (022_Sterivex-1 and 045_Sterivex-2 in St. 35), the complete exclusion of sequencing reads after quality filtering (022_Sterivex-4 in K2), or because replicated sampling was not conducted (022_Sterivex in St. 10).

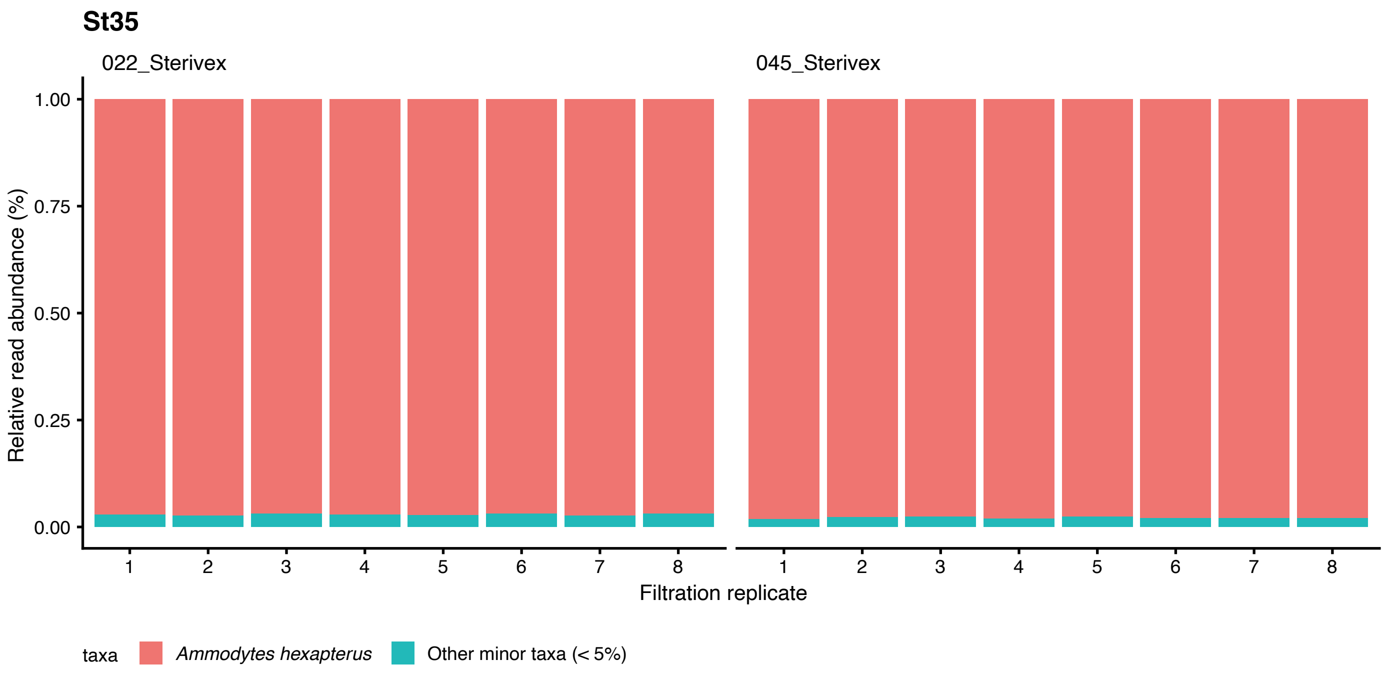

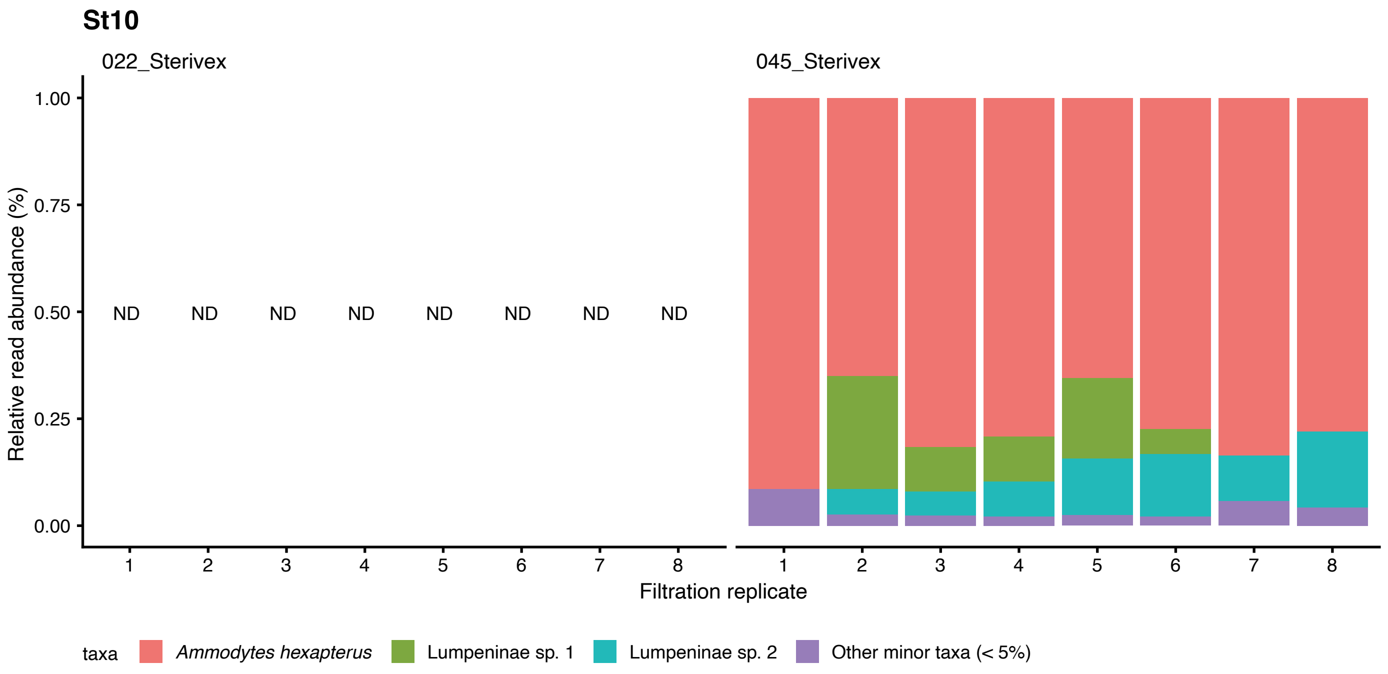

Figure S2 (continued)

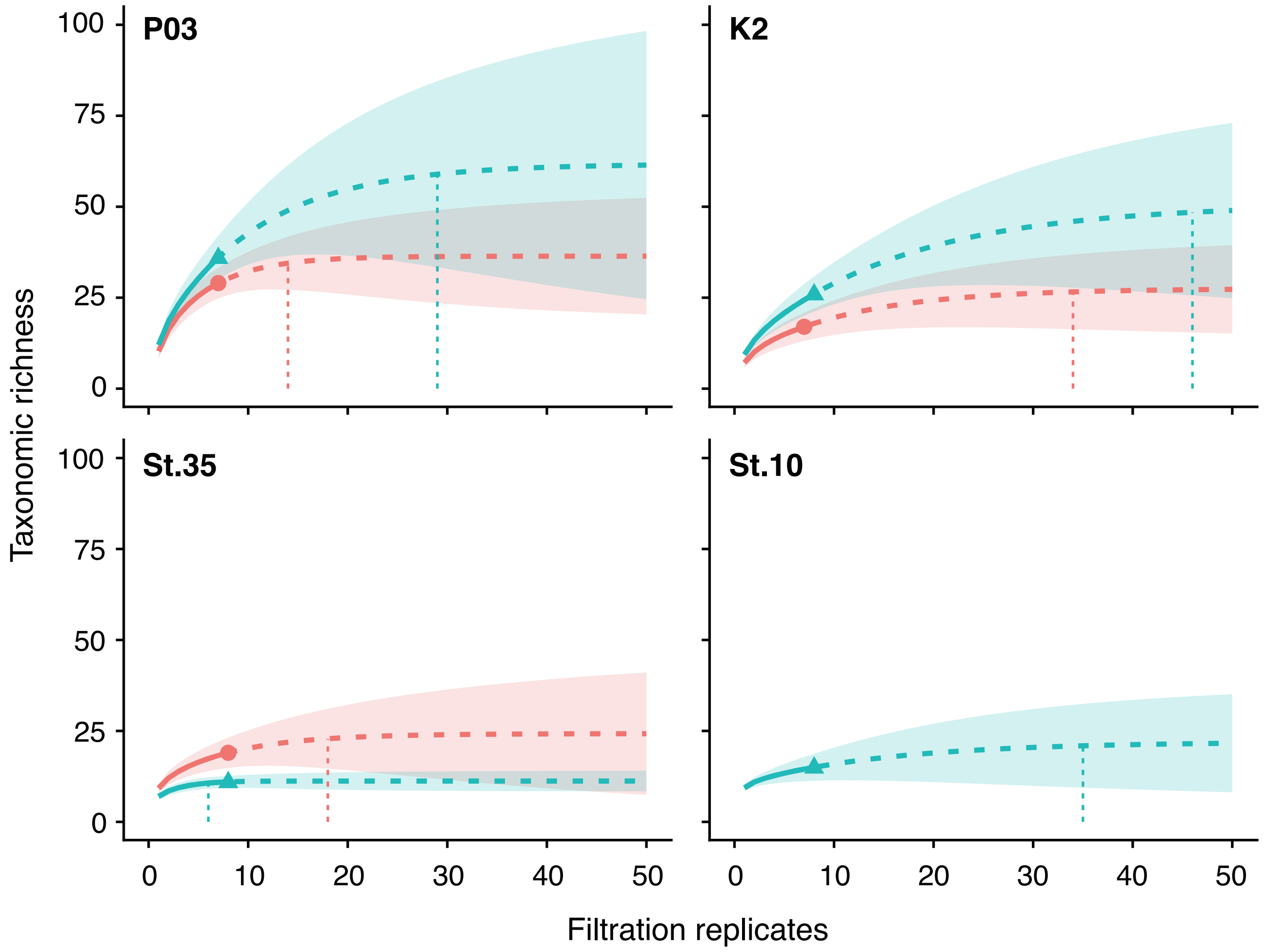

Figure S3 Rarefaction (solid line segment) and extrapolation (broken line segment) curves with 95% confidence intervals (shaded areas) for taxonomic richness derived from data on fish taxonomic composition detected by eDNA metabarcoding with respect to the number of filtration replicates. The color of the line, area, and symbol corresponds to the filter types: 0.22 μm Sterivex-GV (022_Sterivex) and 0.45 μm Sterivex-HV (045_Sterivex) were denoted by red and blue, respectively. The symbols on the lines represent the observed value after aggregating all filtration replicates. Vertical dotted lines below the curves indicate the smallest accumulated filtration volume required to cover ≥95% of the Chao 2 estimator.

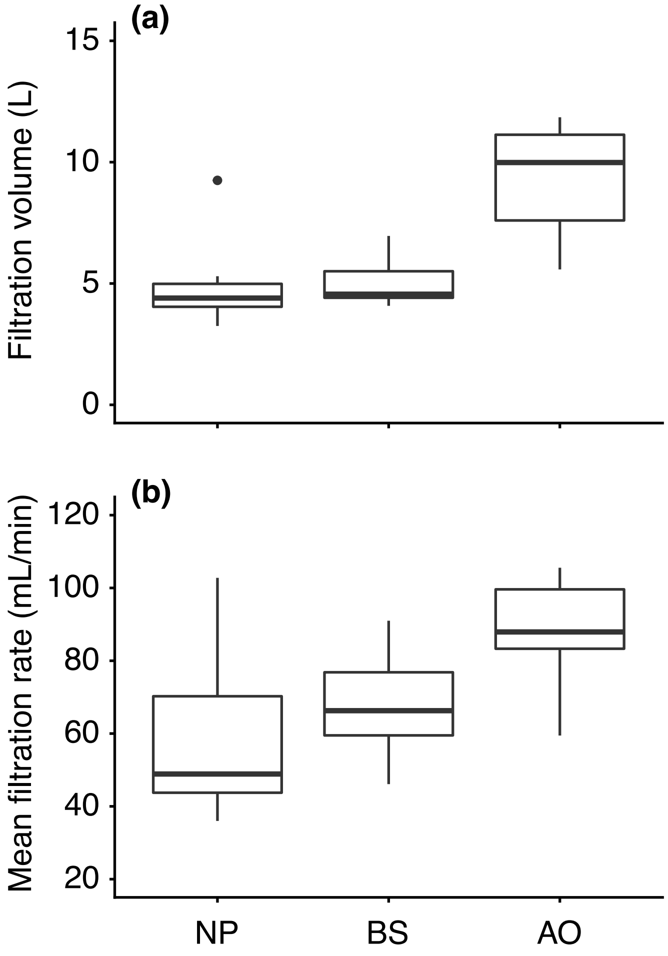

Figure S4 Box plots showing (a) maximum filtration volume per filter (L) and (b) mean filtration speed (mL/min) when using the 0.45 μm Sterivex-HV filter (045_Sterivex) unit for vacuum filtration of seawater collected in the open ocean. NP, BS, and AO denote the Northwestern Pacific Ocean, the Bering Sea, and the Arctic Ocean, respectively. The bold lines in the boxes indicate medians, and the hinges of the boxes indicate the interquartile range (IQR; the first and third quartiles). Whiskers extend to the largest or smallest values no further than 1.5 ×IQR from the hinge. Black dots represent outliers with values greater than 1.5 ×IQR.

Table S1 The number of sequencing reads remained after each step of bioinformatic processing. Detailed information about the procedure used in this study is provided in the manuscript. 022_Srteivex and 045_Sterivex denote 0.22 µm Sterivex-GV and 0.45 µm Sterivex-HV, respectively. The result of P03-045-1 was excluded from the analysis due to an unintended filtration volume. NA indicates not applicable.

Accession No.	Run ID	Library ID	Sample ID	Station	Filter type	Description	Filtration volume (L)	Volume of 2nd PCR product introduced to a sample (µL)	Raw reads	Merge	Quality filtering	Denoising	Chimera removal	Reads those lowest common ancestor was found in first QCauto	Decontamination	Proportional correction to the introduced volume	Reads assigned to actinopterygii after taxnomic modification
DRR400328	RIR005	HKA130	St35-045-NC	St.35	045_Sterivex	Field blank	0.5	4	3622	3018	158	158	157	147	NA	NA	NA
DRR400329	RIR005	HKA131	St35-045-1	St.35	045_Sterivex	Sample replicate	3	2	48086	46917	45935	45935	45921	45921	45627	91520	91446
DRR400330	RIR005	HKA132	St35-045-2	St.35	045_Sterivex	Sample replicate	3	2	57771	56236	55157	55157	55131	55131	54894	110046	109998
DRR400331	RIR005	HKA133	St35-045-3	St.35	045_Sterivex	Sample replicate	3	2	63124	61364	59957	59957	59917	59917	59661	119622	119592
DRR400332	RIR005	HKA134	St35-045-4	St.35	045_Sterivex	Sample replicate	3	2	59139	57686	56597	56597	56578	56578	56365	112912	112870
DRR400333	RIR005	HKA135	St35-045-5	St.35	045_Sterivex	Sample replicate	3	2	59354	57697	56379	56379	56352	56352	56078	112448	112404
DRR400334	RIR005	HKA136	St35-045-6	St.35	045_Sterivex	Sample replicate	3	2	52313	50992	49823	49823	49794	49794	49469	99244	99244
DRR400335	RIR005	HKA137	St35-045-7	St.35	045_Sterivex	Sample replicate	3	2	59422	57900	56564	56564	56537	56537	56256	112822	112802
DRR400336	RIR005	HKA138	St35-045-8	St.35	045_Sterivex	Sample replicate	3	2	48581	47402	46451	46451	46436	46436	46194	92652	92638
DRR400337	RIR005	HKA151	St10-045-NC	St.10	045_Sterivex	Field blank	0.5	4	4800	4006	284	284	280	280	NA	NA	NA
DRR400338	RIR005	HKA152	St10-045-1	St.10	045_Sterivex	Sample replicate	5	2	54885	53408	52236	52236	51912	51911	51671	103536	103520
DRR400339	RIR005	HKA153	St10-045-2	St.10	045_Sterivex	Sample replicate	5	2	43995	42828	41969	41969	41419	41419	41174	82600	82600
DRR400340	RIR005	HKA154	St10-045-3	St.10	045_Sterivex	Sample replicate	5	2	49388	48133	47149	47149	46659	46657	46441	93086	93086
DRR400341	RIR005	HKA155	St10-045-4	St.10	045_Sterivex	Sample replicate	5	2	38498	37598	36861	36861	36416	36416	36114	72538	72498
DRR400342	RIR005	HKA156	St10-045-5	St.10	045_Sterivex	Sample replicate	5	2	43325	42095	41147	41147	40444	40443	40167	80610	80610
DRR400343	RIR005	HKA157	St10-045-6	St.10	045_Sterivex	Sample replicate	5	2	40512	39448	38579	38579	38105	38105	37831	75978	75978
DRR400344	RIR005	HKA158	St10-045-7	St.10	045_Sterivex	Sample replicate	5	2	48399	47063	45974	45974	45503	45502	45253	90762	90762
DRR400345	RIR005	HKA159	St10-045-8	St.10	045_Sterivex	Sample replicate	5	6	111810	108606	105907	105907	104809	104808	104641	69636	69636
DRR400346	HFS001	HKA219	K2-045-NC	K2	045_Sterivex	Field blank	0.5	4	9584	7939	345	345	336	321	NA	NA	NA
DRR400347	HFS001	HKA220	K2-045-1	K2	045_Sterivex	Sample replicate	3	4	149906	146359	139692	139692	139543	138860	138570	138628	135820
DRR400348	HFS001	HKA221	K2-045-2	K2	045_Sterivex	Sample replicate	3	2	149697	146335	144008	144008	143908	143905	143592	287614	285688
DRR400349	HFS001	HKA222	K2-045-3	K2	045_Sterivex	Sample replicate	3	2	141410	138104	134561	134561	134465	134389	134090	268618	204188
DRR400350	HFS001	HKA223	K2-045-4	K2	045_Sterivex	Sample replicate	3	2	81590	79776	77858	77858	77753	77534	77390	154930	109112
DRR400351	HFS001	HKA224	K2-045-5	K2	045_Sterivex	Sample replicate	3	6	97834	95415	87571	87571	87520	86600	86418	57561	56161
DRR400352	HFS001	HKA225	K2-045-6	K2	045_Sterivex	Sample replicate	3	6	56204	54508	48265	48265	48057	47857	47584	31657	31606
DRR400353	HFS001	HKA226	K2-045-7	K2	045_Sterivex	Sample replicate	3	6	63028	61389	54899	54899	54751	54095	53774	35806	34717
DRR400354	HFS001	HKA227	K2-045-8	K2	045_Sterivex	Sample replicate	3	6	89687	87454	79623	79623	79491	78768	78437	52194	42295
DRR400355	HFS001	HKA228	K2-022-NC	K2	022_Sterivex	Field blank	0.5	4	11483	9579	413	413	409	405	NA	NA	NA
DRR400356	HFS001	HKA229	K2-022-1	K2	022_Sterivex	Sample replicate	1.5	6	20246	18517	9525	9525	9495	9138	8973	5950	5825
DRR400357	HFS001	HKA230	K2-022-2	K2	022_Sterivex	Sample replicate	1.5	6	23398	21340	10526	10526	10511	10382	10228	6812	6256
DRR400358	HFS001	HKA231	K2-022-3	K2	022_Sterivex	Sample replicate	1.5	6	18816	16316	4985	4985	4965	4889	4690	3104	3019
DRR400359	HFS001	HKA232	K2-022-4	K2	022_Sterivex	Sample replicate	1.5	6	150	136	39	39	39	39	0	0	0
DRR400360	HFS001	HKA233	K2-022-5	K2	022_Sterivex	Sample replicate	1.5	6	15913	14015	4117	4117	4098	4074	4003	2662	2576
DRR400361	HFS001	HKA234	K2-022-6	K2	022_Sterivex	Sample replicate	1.5	6	28944	26318	15485	15485	15448	15400	15258	10164	9933
DRR400362	HFS001	HKA235	K2-022-7	K2	022_Sterivex	Sample replicate	1.5	6	19845	17341	4602	4602	4597	4544	4357	2882	2752
DRR400363	HFS001	HKA236	K2-022-8	K2	022_Sterivex	Sample replicate	1.5	6	46459	44212	37028	37028	37004	36510	36301	24174	13038
DRR400364	HFS002	HKA237	St35-022-NC	St.35	022_Sterivex	Field blank	0.5	4	18093	14815	855	855	841	841	NA	NA	NA
DRR400365	HFS002	HKA238	St35-022-1	St.35	022_Sterivex	Sample replicate	1.5	2	115390	113284	109272	109272	109246	109238	108885	218298	217618
DRR400366	HFS002	HKA239	St35-022-2	St.35	022_Sterivex	Sample replicate	1.5	2	88192	86396	82202	82202	82187	82176	81949	164108	163944
DRR400367	HFS002	HKA240	St35-022-3	St.35	022_Sterivex	Sample replicate	1.5	2	95189	93304	88803	88803	88773	88766	88580	177370	176780
DRR400368	HFS002	HKA241	St35-022-4	St.35	022_Sterivex	Sample replicate	1.5	2	136791	134691	130883	130883	130856	130847	130670	261586	261018
DRR400369	HFS002	HKA242	St35-022-5	St.35	022_Sterivex	Sample replicate	1.5	2	137715	135655	132048	132048	132009	131979	131801	263766	263288
DRR400370	HFS002	HKA243	St35-022-6	St.35	022_Sterivex	Sample replicate	1.5	2	133925	131986	128166	128166	128136	128123	127887	256024	255424
DRR400371	HFS002	HKA244	St35-022-7	St.35	022_Sterivex	Sample replicate	1.5	2	123792	121709	117867	117867	117848	117845	117629	235506	235258
DRR400372	HFS002	HKA245	St35-022-8	St.35	022_Sterivex	Sample replicate	1.5	2	115531	113738	109961	109961	109939	109914	109673	219490	219048
DRR400373	HFS002	HKA246	P03-022-NC	P03	022_Sterivex	Field blank	0.5	4	18028	14753	224	224	216	202	NA	NA	NA
DRR400374	HFS002	HKA247	P03-022-1	P03	022_Sterivex	Sample replicate	3	6	56404	53849	34199	34199	32256	32187	32057	21350	21350
DRR400375	HFS002	HKA248	P03-022-2	P03	022_Sterivex	Sample replicate	3	4	17437	15511	3514	3514	3512	2983	2794	2770	2617
DRR400376	HFS002	HKA249	P03-022-3	P03	022_Sterivex	Sample replicate	3	4	18704	17202	6454	6454	6449	6412	6065	6060	5905
DRR400377	HFS002	HKA250	P03-022-4	P03	022_Sterivex	Sample replicate	3	4	26974	25262	13512	13512	12902	12605	12343	12361	12361

Table S1. Continued

Accession No.	Run ID	Library ID	Sample ID	Station	Filter type	Description	Filtration volume (L)	Volume of 2nd PCR product introduced to a sample (μL)	Raw reads	Merge	Quality filtering	Denoising	Chimera removal	Reads those lowest common ancestor was found in first QCauto	Decontamination	Proportional correction to the introduced volume	Reads assigned to actinopterygii after taxnomic modification
DRR400378	HFS002	HKA251	P03-022-5	P03	022_Sterivex	Sample replicate	3	6	25217	21989	2454	2454	2452	2386	2199	1419	1419
DRR400379	HFS002	HKA252	P03-022-6	P03	022_Sterivex	Sample replicate	3	2	176454	174370	169991	169991	168624	168567	168382	336892	336830
DRR400380	HFS002	HKA253	P03-022-7	P03	022_Sterivex	Sample replicate	3	4	163468	160995	151633	151633	150884	150833	150648	150612	150612
DRR400381	HFS002	HKA254	P03-045-NC	P03	045_Sterivex	Field blank	0.5	4	21495	17730	220	220	218	218	NA	NA	NA
DRR400382	HFS002	HKA255	P03-045-1	P03	045_Sterivex	Sample replicate	4	6	35537	32505	10161	10161	10155	10148	9943	NA	NA
DRR400383	HFS002	HKA256	P03-045-2	P03	045_Sterivex	Sample replicate	3	6	29844	26906	11410	11410	11400	10988	10719	7086	7086
DRR400384	HFS002	HKA257	P03-045-3	P03	045_Sterivex	Sample replicate	3	6	24670	21634	5506	5506	5501	5418	5178	3416	3416
DRR400385	HFS002	HKA258	P03-045-4	P03	045_Sterivex	Sample replicate	3	6	24809	22016	3069	3069	2782	2682	2416	1605	1563
DRR400386	HFS002	HKA259	P03-045-5	P03	045_Sterivex	Sample replicate	3	6	129854	126140	113167	113167	113163	111619	111310	74161	73611
DRR400387	HFS002	HKA260	P03-045-6	P03	045_Sterivex	Sample replicate	3	2	275615	272073	266283	266283	265149	265140	264710	529650	529454
DRR400388	HFS002	HKA261	P03-045-7	P03	045_Sterivex	Sample replicate	3	4	189007	185404	175913	175913	174699	173479	173024	173049	172913
DRR400389	HFS002	HKA262	P03-045-8	P03	045_Sterivex	Sample replicate	3	6	190597	185960	171910	171910	171869	170112	169728	113009	112929
DRR400390	RIR005	NTC101	NTC101	NA	NA	No-template control for PCR	NA	4	6121	5093	285	285	284	284	NA	NA	NA
DRR400391	RIR005	NTC102	NTC102	NA	NA	No-template control for PCR	NA	4	5466	4561	127	127	124	124	NA	NA	NA
DRR400392	RIR005	NTC103	NTC103	NA	NA	No-template control for PCR	NA	4	6978	5691	414	414	409	409	NA	NA	NA
DRR400393	HFS001	NTC201	NTC201	NA	NA	No-template control for PCR	NA	4	10251	8323	299	299	280	279	NA	NA	NA
DRR400394	HFS002	NTC202	NTC202	NA	NA	No-template control for PCR	NA	4	19678	15753	118	118	116	114	NA	NA	NA
DRR400395	HFS002	NTC203	NTC203	NA	NA	No-template control for PCR	NA	4	16176	13227	36	36	36	35	NA	NA	NA

Table S2 Summary of taxonomic assignment results obtained by the QCauto algorithm (Tanabe and Toju 2013) and modifications based on distribution knowledge. Candidate species names are consistent with scientific names adopted in the NCBI taxonomy database.

ID	Candidate species	Order	Family	Genus	Lowest Common Ancestor	Common name	Number of ASV consolidated	Range of pairwise distances within OTU (mean ± SD)	Pairwise distances between other OTUs (mean ± SD)
denoised_008	*Engraulis japonicus*	Clupeiformes	Engraulidae	*Engraulis*	*Engraulis japonicus*	Japanese anchovy	13	0.6–2.3 (0.9±0.5)	22.2–38.2 (27.9±3.4)
denoised_003	*Sardinops melanostictus/Sardinops sagax*	Clupeiformes	Clupeidae	*Sardinops*	*Sardinops sagax* †	Pacific sardine	29	0.6–2.9 (0.9±0.6)	21.7–35.6 (28.7±2.5)
denoised_091	*Oncorhynchus keta/Oncorhynchus nerka*	Salmoniformes	Salmonidae	*Oncorhynchus*	*Oncorhynchus sp.*	Salmonids	2	0.6 (0.6)	16.6–34.2 (25.3±3.4)
denoised_254	*Leuroglossus schmidti*	Argentiniformes	Bathylagidae	*Leuroglossus*	*Leuroglossus schmidti*	Northern smooth-tongue	1	NA	15.4–31.8 (21.2±4)
denoised_600	*Mallotus villosus*	Osmeriformes	Salangidae	*Mallotus*	*Mallotus villosus*	Capelin	1	NA	17.2–34.8 (23.5±3.4)
denoised_198	*Cyclothone alba*	Stomiiformes	Gonostomatidae	*Cyclothone*	*Cyclothone alba*	Bristlemouth	2	0.6 (0.6)	18.7–41.6 (31.9±3.3)
denoised_026	*Cyclothone pseudopallida*	Stomiiformes	Gonostomatidae	*Cyclothone*	*Cyclothone pseudopallida*	Slender bristlemouth	5	0.6 (0.6±0)	18.7–37.9 (28.4±3.2)
denoised_015	*Woodsia nonsuchae/Vinciguerria nimbaria*	Stomiiformes	Phosichthyidae		Phosichthyidae sp. 1	Lightfishes	5	0.6 (0.6±0)	0.6–35.8 (28.2±4.1)
denoised_344	*Woodsia nonsuchae/Vinciguerria nimbaria/Vinciguerria* sp. CBM:ZF:14736	Stomiiformes	Phosichthyidae		Phosichthyidae sp. 2	Lightfishes	2	1.2 (1.2)	0.6–36.1 (28.2±4.1)
denoised_047	*Tactostoma macropus*	Stomiiformes	Stomiidae	*Tactostoma*	*Tactostoma macropus*	Longfin dragonfish	3	0.6 (0.6±0)	16.3–33.3 (23.9±3.5)
denoised_006	*Diplophos* sp. HUMZ:222608/*Diplophos orientalis*	Stomiiformes	Diplophidae	*Diplophos*	*Diplophos* sp. 1	Bristlemouths	5	0.6 (0.6±0)	0.6–33.3 (22.6±4.5)
denoised_108	*Diplophos* sp. HUMZ:222608/*Diplophos taenia/Diplophos orientalis*	Stomiiformes	Diplophidae	*Diplophos*	*Diplophos* sp. 2	Bristlemouths	8	1.2–3.5 (1.5±0.9)	0.6–30.8 (22.5±4.4)
denoised_184	*Diplophos* sp. MM-1999/*Diplophos taenia*	Stomiiformes	Diplophidae	*Diplophos*	*Diplophos* sp. 3	Bristlemouths	2	0.6 (0.6)	6.4–33.7 (24.4±4.1)
denoised_049	*Bolinichthys pyrsobolus*	Myctophiformes	Myctophidae	*Bolinichthys*	*Bolinichthys pyrsobolus*	Fiery lanternfish	6	0.6–1.7 (0.8±0.5)	9.2–35.7 (26.5±5.9)
denoised_520	*Diaphus anderseni*	Myctophiformes	Myctophidae	*Diaphus*	*Diaphus anderseni*	Andersen's lantern fish	1	NA	11.1–34.2 (26.4±4.7)
denoised_397	*Diaphus parri/Diaphus* sp. CBM:ZF:14790	Myctophiformes	Myctophidae	*Diaphus*	*Diaphus* sp.	Lanternfishes	1	NA	9.3–33 (25.5±4.6)
denoised_238	*Diaphus theta*	Myctophiformes	Myctophidae	*Diaphus*	*Diaphus theta*	California headlightfish	1	NA	9.3–35.3 (25.9±5.0)
denoised_055	*Diogenichthys atlanticus/Diogenichthys* sp. CBM:ZF:14787/*Diogenichthys laternatus*	Myctophiformes	Myctophidae	*Diogenichthys*	*Diogenichthys* sp.	Lanternfishes	2	0.6 (0.6)	18.8–36.2 (26.6±3.4)
denoised_429	*Hygophum reinhardtii*	Myctophiformes	Myctophidae	*Hygophum*	*Hygophum reinhardtii*	Reinhardt's lantern fish	1	NA	18.8–35 (26.5±3.6)
denoised_018	*Lampadena luminosa*	Myctophiformes	Myctophidae	*Lampadena*	*Lampadena luminosa*	Luminous lanternfish	3	0.6 (0.6±0)	0.6–38 (26.8±5.8)
denoised_005	*Dasyscopelus asper/Myctophum spinosum/Myctophum lychnobium*	Myctophiformes	Myctophidae		Myctophidae sp. 1	Lanternfishes	16	0.6–2.3 (0.9±0.5)	17.4–36.9 (26.4±4.2)
denoised_017	*Symbolophorus californiensis/Symbolophorus evermanni/Loweina terminata*	Myctophiformes	Myctophidae		Myctophidae sp. 2	Lanternfishes	4	0.6 (0.6±0)	15.4–39.8 (29.6±4.7)
denoised_337	*Diaphus luetkeni/Lampadena luminosa*	Myctophiformes	Myctophidae		Myctophidae sp. 3	Lanternfishes	3	1.2 (1.2±0)	0.6–37.8 (27.1±5.8)
denoised_418	*Symbolophorus californiensis/Tarletonbeania crenularis/Loweina terminata*	Myctophiformes	Myctophidae		Myctophidae sp. 4	Lanternfishes	1	NA	2.3–34.2 (24.9±4.5)
denoised_377	*Stenobrachius leucopsarus/Stenobrachius nannochir/Lampanyctus* sp. CBM:ZF:14792/*Lampanyctus simulator*	Myctophiformes	Myctophidae		Myctophidae sp. 5	Lanternfishes	1	NA	1.2–35.5 (25.4±6.5)
denoised_107	*Notoscopelus japonicus*	Myctophiformes	Myctophidae	*Notoscopelus*	*Notoscopelus japonicus*	Japanese lanternfish	2	0.6 (0.6)	6.3–33.9 (25.2±5.8)
denoised_028	*Stenobrachius leucopsarus*	Myctophiformes	Myctophidae	*Stenobrachius*	*Stenobrachius leucopsarus*	Northern lampfish	4	0.6 (0.6±0)	1.2–35.1 (24.8±6.2)
denoised_034	*Stenobrachius leucopsarus/Stenobrachius nannochir*	Myctophiformes	Myctophidae	*Stenobrachius*	*Stenobrachius sp.*	Lanternfishes	8	0.6–2.9 (1.2±0.9)	1.2–34.6 (24.8±6.4)
denoised_004	*Symbolophorus californiensis*	Myctophiformes	Myctophidae	*Symbolophorus*	*Symbolophorus californiensis*	Bigfin lanternfish	36	0.6–1.8 (1±0.4)	2.3–34.4 (25.4±4.4)
denoised_295	*Boreogadus saida*	Gadiformes	Gadidae	*Boreogadus*	*Boreogadus saida*	Arctic cod	1	NA	1.8–39.8 (27.3±6.8)
denoised_456	*Eleginus gracilis*	Gadiformes	Gadidae	*Eleginus*	*Eleginus gracilis*	Saffron cod	1	NA	3.5–39.5 (27.7±6.6)
denoised_345	*Gadus chalcogrammus/Arctogadus glacialis*	Gadiformes	Gadidae		Gadidae sp.	Cods	1	NA	0.6–41.3 (27.6±6.8)
denoised_357	*Gadus chalcogrammus*	Gadiformes	Gadidae	*Gadus*	*Gadus chalcogrammus*	Walleye pollock	1	NA	1.2–39.8 (27.2±6.8)
denoised_094	*Gadus chalcogrammus/Gadus macrocephalus*	Gadiformes	Gadidae	*Gadus*	*Gadus* sp.	Cods	2	0.6 (0.6)	0.6–41.6 (27.7±6.9)
denoised_242	*Beryx splendens/Scopeloberyx robustus/Poromitra* sp. CBM:ZF:14725	Beryciformes			Beryciformes sp.	Whalefishes and others	2	0.6 (0.6)	15.3–33.7 (23.4±3.4)
denoised_558	*Rhinogobius brunneus*	Gobiiformes	Gobiidae	*Rhinogobius*	*Rhinogobius brunneus*	Amur goby	1	NA	17.4–34.2 (25.3±3.6)
denoised_078	*Cheilopogon agoo/Cheilopogon agoo agoo*	Beloniformes	Exocoetidae	*Cheilopogon*	*Cheilopogon agoo*	Japanese flyingfish	2	0.6 (0.6)	1.2–30.4 (20.7±7.4)

†*Sardinops melanostictus* is regarded as the same species as *S. sagax* in FishBase.

Table S2. Continued

ID	Candidate species	Order	Family	Genus	Lowest Common Ancestor	Common name	Number of ASV consolidated	Range of pairwise distances within OTU (mean ± SD)	Pairwise distances between other OTUs (mean ± SD)
denoised_067	*Cheilopogon atrisignis/Cheilopogon cyanopterus/Cheilopogon suttoni*	Beloniformes	Exocoetidae	*Cheilopogon*	*Cheilopogon* sp.	Flyingfishes	1	NA	0.6–30.6 (21.3±7.3)
denoised_021	*Exocoetus volitans/Cheilopogon unicolor/Cheilopogon arcticeps/Cheilopogon doederleinii*	Beloniformes	Exocoetidae		Exocoetidae sp. 1	Flyingfishes	4	0.6 (0.6±0)	0.6–31.5 (21±7.6)
denoised_019	*Cheilopogon unicolor/Cheilopogon arcticeps/Cheilopogon doederleinii/Hirundichthys speculiger/Hirundichthys oxycephalus*	Beloniformes	Exocoetidae		Exocoetidae sp. 2	Flyingfishes	3	0.6 (0.6±0)	0.6–31.3 (20.7±7.8)
denoised_053	*Cypselurus hiraii/Cheilopogon pinnatibarbatus japonicus/Cheilopogon unicolor/Cheilopogon arcticeps/Cheilopogon doederleinii/Cheilopogon antoncichi/Cheilopogon spilonotopterus/Cypselurus starksi/Cypselurus poecilopterus/Cypselurus naresii/Cheilopogon cyanopterus/Cypselurus opisthopus/Cypselurus oligolepis*	Beloniformes	Exocoetidae		Exocoetidae sp. 3	Flyingfishes	1	NA	0.6–30.4 (20.8±7.6)
denoised_105	*Cypselurus hiraii/Cheilopogon pinnatibarbatus japonicus/Exocoetus volitans/Cheilopogon doederleinii/Cheilopogon unicolor/Cheilopogon arcticeps/Prognichthys sealei/Cheilopogon antoncichi/Cheilopogon spilonotopterus/Cypselurus starksi/Cheilopogon exsiliens/Cypselurus naresii/Hirundichthys speculiger/Cheilopogon cyanopterus/Cypselurus opisthopus/Hirundichthys oxycephalus/Cypselurus oligolepis/Hirundichthys rondeletii*	Beloniformes	Exocoetidae		Exocoetidae sp. 4	Flyingfishes	4	1.2 (1.2±0)	0.6–30.4 (20.6±7.6)
denoised_230	*Cypselurus hiraii/Cheilopogon pinnatibarbatus japonicus/Exocoetus volitans/Cheilopogon doederleinii/Cheilopogon unicolor/Cheilopogon arcticeps/Prognichthys sealei/Cheilopogon antoncichi/Cheilopogon spilonotopterus/Cypselurus starksi/Cheilopogon exsiliens/Cypselurus naresii/Hirundichthys speculiger/Cheilopogon cyanopterus/Cypselurus opisthopus/Hirundichthys oxycephalus/Cypselurus oligolepis/Cheilopogon atrisignis/Hirundichthys rondeletii*	Beloniformes	Exocoetidae		Exocoetidae sp. 5	Flyingfishes	2	1.8 (1.8)	1.2–31.5 (21±7.2)
denoised_479	*Cheilopogon atrisignis/Cheilopogon cyanopterus/Cypselurus angusticeps/Cheilopogon suttoni*	Beloniformes	Exocoetidae		Exocoetidae sp. 6	Flyingfishes	1	NA	0.6–30.6 (21.7±7.3)
denoised_464	*Cypselurus hiraii/Cheilopogon pinnatibarbatus japonicus/Cheilopogon unicolor/Cheilopogon arcticeps/Cheilopogon doederleinii/Cheilopogon antoncichi/Cheilopogon spilonotopterus/Cypselurus starksi/Cypselurus naresii/Cheilopogon cyanopterus/Cypselurus opisthopus/Cypselurus oligolepis*	Beloniformes	Exocoetidae		Exocoetidae sp. 7	Flyingfishes	1	NA	0.6–30.9 (21.1±7.7)
denoised_543	*Cypselurus hiraii/Cheilopogon pinnatibarbatus japonicus/Exocoetus volitans/Cheilopogon unicolor/Cheilopogon arcticeps/Cheilopogon doederleinii/Cheilopogon antoncichi/Cheilopogon spilonotopterus/Cypselurus starksi/Cypselurus naresii/Cheilopogon cyanopterus/Cypselurus opisthopus/Cypselurus oligolepis/Cheilopogon atrisignis*	Beloniformes	Exocoetidae		Exocoetidae sp. 8	Flyingfishes	1	NA	1.2–31.8 (21.6±7.8)
denoised_399	*Hemiramphus convexus/Oxyporhamphus micropterus micropterus*	Beloniformes	Hemiramphidae		Hemiramphidae sp.	Flyingfishes	1	NA	13.4–31.9 (22.5±4.9)
denoised_065	*Cololabis saira*	Beloniformes	Belonidae	*Cololabis*	*Cololabis saira*	Pacific saury	2	0.6 (0.6)	14.9–30.9 (22.4±3.6)

Table S2. Continued

ID	Candidate species	Order	Family	Genus	Lowest Common Ancestor	Common name	Number of ASV consolidated	Range of pairwise distances within OTU (mean ± SD)	Pairwise distances between other OTUs (mean ± SD)
denoised_294	*Platichthys stellatus/Limanda aspera/Acanthopsetta nadeshnyi/Hippoglossoides elassodon/Hippoglossoides robustus/Limanda sakhalinensis*	Pleuronectiformes	Pleuronectidae		Pleuronectidae sp. 1	Righteye flounders	1	NA	0.6–34.2 (22.1±4.6)
denoised_494	*Limanda aspera/Hippoglossus stenolepis/Acanthopsetta nadeshnyi/Hippoglossoides elassodon/Hippoglossoides robustus/Limanda sakhalinensis*	Pleuronectiformes	Pleuronectidae		Pleuronectidae sp. 2	Righteye flounders	1	NA	0.6–34.4 (22.1±4.6)
denoised_575	*Platichthys stellatus/Glyptocephalus zachirus*	Pleuronectiformes	Pleuronectidae		Pleuronectidae sp. 3	Righteye flounders	1	NA	1.2–32.6 (22±4.4)
denoised_387	*Japonolaeops dentatus/Arnoglossus yamanakai*	Pleuronectiformes	Bothidae		Bothidae sp.	Lefteye flounders	1	NA	12.1–33.5 (21.8±4.0)
denoised_311	*Bothus pantherinus*	Pleuronectiformes	Bothidae	*Bothus*	*Bothus pantherinus*	Leopard flounder	1	NA	12.1–31.8 (23.3±3.1)
denoised_057	*Diplospinus multistriatus*	Scombriformes	Gempylidae	*Diplospinus*	*Diplospinus multistriatus*	Striped escolar	2	0.6 (0.6)	12.3–35.5 (21.7±4.4)
denoised_037	*Gempylus serpens*	Scombriformes	Gempylidae	*Gempylus*	*Gempylus serpens*	Snake mackerel	4	0.6 (0.6±0)	9.4–29 (19.4±4.3)
denoised_012	*Katsuwonus pelamis*	Scombriformes	Scombridae	*Katsuwonus*	*Katsuwonus pelamis*	Skipjack tuna	4	0.6 (0.6±0)	0.6–30.8 (18.8±5.0)
denoised_009	*Scomber japonicus/Scomber australasicus*	Scombriformes	Scombridae	*Scomber*	*Scomber* sp.	Mackerels	6	0.6–1.8 (0.8±0.5)	9.5–32.6 (21.7±4.1)
denoised_176	*Katsuwonus pelamis/Auxis rochei/Euthynnus affinis*	Scombriformes	Scombridae		Thunnini sp.	Mackerels	2	1.2 (1.2)	0.6–30.8 (18.6±5)
denoised_062	*Dysalotus alcocki/Pseudoscopelus* sp. HUMZ:220812/*Dysalotus* sp. CBM:ZF:14697/*Pseudoscopelus obtusifrons/Pseudoscopelus sagamianus*	Scombriformes	Chiasmodontidae		Chiasmodontidae sp.	Black swallowers	3	0.6–1.2 (0.9±0.4)	0.6–33.7 (22.2±4.5)
denoised_101	*Dysalotus* sp. CBM:ZF:14697	Scombriformes	Chiasmodontidae	*Dysalotus*	*Dysalotus* sp.	Black swallowers	1	NA	0.6–33.9 (22.2±4.6)
denoised_001	*Ammodytes hexapterus*	Uranoscopiformes	Ammodytidae	*Ammodytes*	*Ammodytes hexapterus*	Pacific sand lance	10	0.6–1.2 (0.9±0.3)	0.6–28.6 (19.5±4.5)
denoised_060	*Ammodytes personatus/Ammodytes hexapterus*	Uranoscopiformes	Ammodytidae	*Ammodytes*	*Ammodytes* sp.	Sand lances	67	1.2–1.8 (1.2±0.1)	0.6–29.3 (19.5±4.6)
denoised_499	*Calotomus carolinus/Calotomus spinidens*	Labriformes	Labridae	*Calotomus*	*Calotomus* sp.	Wrasses	1	NA	19.2–34.2 (26±3.5)
denoised_421	*Thalassoma cupido*	Labriformes	Labridae	*Thalassoma*	*Thalassoma cupido*	Cupid wrasse	1	NA	17.6–34.9 (26.1±3.3)
denoised_024	*Epinephelus areolatus*	Perciformes	Serranidae	*Epinephelus*	*Epinephelus areolatus*	Areolate grouper	5	0.6 (0.6±0)	0.6–34.7 (24.4±4.4)
denoised_474	*Epinephelus areolatus/Epinephelus chlorostigma*	Perciformes	Serranidae	*Epinephelus*	*Epinephelus* sp.	Sea basses	1	NA	0.6–35.2 (24.9±4.5)
denoised_389	*Pterycombus petersii/Eumegistus illustris/Taractes asper*	Scombriformes	Bramidae		Bramidae sp. 1	Pomfrets	1	NA	10.8–34.4 (22.4±4.0)
denoised_606	*Taractes asper/Brama japonica/Brama dussumieri*	Scombriformes	Bramidae		Bramidae sp. 2	Pomfrets	1	NA	10.8–35.1 (25.4±3.9)
denoised_020	*Katsuwonus pelamis/Thunnus alalunga/Auxis rochei/Thunnus orientalis/Euthynnus affinis/Cubiceps baxteri/Thunnus obesus/Thunnus albacares/Thunnus tonggol/Auxis thazard*	Scombriformes			Scombriformes sp.	Tunas and others	2	0.6 (0.6)	1.8–32.6 (19.4±5)
denoised_090	*Parapristipoma trilineatum*	Lutjaniformes	Haemulidae	*Parapristipoma*	*Parapristipoma trilineatum*	Chicken grunt	2	0.6 (0.6)	18.5–38.3 (25.4±4.1)
denoised_519	*Gymnelus hemifasciatus/Gymnelus andersoni*	Perciformes	Zoarcidae	*Gymnelus*	*Gymnelus* sp.	Eelpouts	1	NA	8.2–32 (22.2±6.0)
denoised_531	*Lycodes palearis*	Perciformes	Zoarcidae	*Lycodes*	*Lycodes palearis*	Wattled eelpout	1	NA	2.4–33.7 (21±6.7)
denoised_526	*Lycodes raridens*	Perciformes	Zoarcidae	*Lycodes*	*Lycodes raridens*	Marbled eelpout	1	NA	1.8–31.6 (20.7±6.8)
denoised_419	*Lycodes raridens/Lycodes brevipes/Lycodes reticulatus/Lycodes rossi*	Perciformes	Zoarcidae	*Lycodes*	*Lycodes* sp.	Eelpouts	1	NA	1.8–33.2 (20.1±6.7)
denoised_426	*Leptoclinus maculatus*	Perciformes	Stichaeidae	*Leptoclinus*	*Leptoclinus maculatus*	Daubed Shanny	1	NA	2.4–33.8 (20±7.5)
denoised_014	*Anisarchus medius/Poroclinus rothrocki*	Perciformes	Stichaeidae		Lumpeninae sp. 1	Pricklebacks	1	NA	0.6–30.3 (19.5±7.4)
denoised_011	*Leptoclinus maculatus/Lumpenus fabricii/Lumpenus sagitta*	Perciformes	Stichaeidae		Lumpeninae sp. 2	Pricklebacks	6	0.6–1.2 (0.7±0.3)	2.9–33.7 (20±7.5)
denoised_127	*Eumesogrammus praecisus/Anisarchus medius/Poroclinus rothrocki*	Perciformes	Stichaeidae		Stichaeidae sp. 1	Pricklebacks	4	1.8 (1.8±0)	0.6–30.5 (19.4±7.2)
denoised_209	*Eumesogrammus praecisus/Anisarchus medius*	Perciformes	Stichaeidae		Stichaeidae sp. 2	Pricklebacks	1	NA	0.6–31.5 (20.1±7.5)
denoised_375	*Lumpenella longirostris/Eumesogrammus praecisus/Leptoclinus maculatus/Anisarchus medius/Poroclinus rothrocki*	Perciformes	Stichaeidae		Stichaeidae sp. 3	Pricklebacks	1	NA	0.6–30.5 (19.6±7.3)
denoised_524	*Gasterosteus aculeatus/Gasterosteus aculeatus aculeatus*	Perciformes	Gasterosteidae	*Gasterosteus*	*Gasterosteus aculeatus*	Three-spined stickleback	1	NA	9.4–35.2 (21.7±5.3)
denoised_356	*Hexagrammos otakii/Pleurogrammus monopterygius*	Perciformes	Hexagrammidae		*Hexagrammidae* sp.	Greenlings	1	NA	6.5–31.6 (20.6±5.9)
denoised_506	*Ulcina olrikii*	Perciformes	Agonidae	*Ulcina*	*Ulcina olrikii*	Arctic alligatorfish	1	NA	7.7–32.5 (22.4±6.1)

Table S2. Continued

ID	Candidate species	Order	Family	Genus	Lowest Common Ancestor	Common name	Number of ASV consolidated	Range of pairwise distances within OTU (mean ± SD)	Pairwise distances between other OTUs (mean ± SD)
denoised_240	*Microcottus sellaris/Myoxocephalus scorpius/Myoxocephalus quadricornis*	Perciformes	Cottidae		Cottidae sp.	Sculpins	1	NA	0.6–30.4 (20.4±6.1)
denoised_271	*Gymnocanthus tricuspis*	Perciformes	Cottidae	*Gymnocanthus*	*Gymnocanthus tricuspis*	Arctic staghorn sculpin	1	NA	3.5–30.9 (20±5.9)
denoised_424	*Myoxocephalus stelleri/Myoxocephalus polyacanthocephalus/Myoxocephalus scorpius/Myoxocephalus jaok/Myoxocephalus quadricornis*	Perciformes	Cottidae	*Myoxocephalus*	*Myoxocephalus* sp.	Sculpins	1	NA	0.6–30.4 (20.3±6.1)
denoised_093	*Aptocyclus ventricosus/Lethotremus awae*	Perciformes	Cyclopteridae		Cyclopteridae sp.	Lumpfishes	2	0.6 (0.6)	8.9–33 (21.3±6.3)
denoised_508	*Liparis gibbus*	Perciformes	Liparidae	*Liparis*	*Liparis gibbus*	Variegated snailfish	1	NA	8.9–32.5 (23±5.4)
denoised_151	*Abudefduf sexfasciatus/Abudefduf vaigiensis*	Perciformes	Pomacentridae	*Abudefduf*	*Abudefduf* sp.	Damselfishes	3	0.6 (0.6±0)	13.4–32 (22.4±4.3)
denoised_257	*Erythrocles schlegelii/Emmelichthys struhsakeri*	Perciformes	Emmelichthyidae		Emmelichthyidae sp.	Rovers	1	NA	14.4–35 (21.9±4.4)
denoised_002	*Gigantactis vanhoeffeni/Gigantactis elsmani*	Lophiiformes	Gigantactinidae	*Gigantactis*	*Gigantactis* sp.	Whipnose anglers	58	0.6–3 (0.7±0.3)	20.5–36.3 (28.7±3.8)
denoised_042	*Canthidermis maculata*	Tetraodontiformes	Balistidae	*Canthidermis*	*Canthidermis maculata*	Spotted oceanic triggerfish	2	0.6 (0.6)	12.7–35.5 (24±4.8)
denoised_051	*Diodon hystrix/Diodon eydouxii*	Tetraodontiformes	Diodontidae	*Diodon*	*Diodon* sp.	Porcupinefishes	2	0.6 (0.6)	17.2–28.3 (23±2.8)

Table S3 Comparison of taxonomic composition detected as eDNA from two different filters having different pore sizes (022_Sterivex: 0.22 µm Sterivex-GV and 045_Sterivex: 0.45 µm Sterivex-HV) at P03 in the subtropical gyre of the northwestern Pacific Ocean. The numbers in the table indicate the frequency of occurrence. ND indicates that the taxon was not detected in the sample.

Order	Family	Lowest common ancestor	Common name	022_Sterivex	045_Sterivex	Presence
Clupeiformes	Engraulidae	*Engraulis japonicus*	Japanese anchovy	2	5	both
Stomiiformes	Gonostomatidae	*Cyclothone alba*	Bristlemouth	3	ND	022_Sterivex
		Cyclothone pseudopallida	Slender bristlemouth	3	4	both
	Phosichthyidae	Phosichthyidae sp. 1	Lightfishes	5	6	both
		Phosichthyidae sp. 2	Lightfishes	1	ND	022_Sterivex
	Diplophidae	*Diplophos* sp. 1	Bristlemouths	6	6	both
		Diplophos sp. 2	Bristlemouths	3	4	both
		Diplophos sp. 3	Bristlemouths	1	ND	022_Sterivex
Myctophiformes	Myctophidae	*Bolinichthys pyrsobolus*	Fiery lanternfish	2	ND	022_Sterivex
		Diaphus anderseni	Andersen's lantern fish	ND	1	045_Sterivex
		Diaphus sp.	Lanternfishes	ND	1	045_Sterivex
		Diogenichthys sp.	Lanternfishes	ND	1	045_Sterivex
		Hygophum reinhardtii	Reinhardt's lantern fish	1	ND	022_Sterivex
		Lampadena luminosa	Luminous lanternfish	5	4	both
		Myctophidae sp. 1	Lanternfishes	4	3	both
		Myctophidae sp. 2	Lanternfishes	4	2	both
		Myctophidae sp. 3	Lanternfishes	ND	1	045_Sterivex
		Stenobrachius sp.	Lanternfishes	1	ND	022_Sterivex
Beryciformes		Beryciformes sp.	Whalefishes and others	ND	1	045_Sterivex
Gobiiformes	Gobiidae	*Rhinogobius brunneus*	Amur goby	ND	1	045_Sterivex
Beloniformes	Exocoetidae	*Cheilopogon agoo*	Japanese flyingfish	ND	2	045_Sterivex
		Cheilopogon sp.	Flyingfishes	2	ND	022_Sterivex
		Exocoetidae sp. 1	Flyingfishes	6	1	both
		Exocoetidae sp. 2	Flyingfishes	1	1	both
		Exocoetidae sp. 3	Flyingfishes	ND	2	045_Sterivex
		Exocoetidae sp. 4	Flyingfishes	2	2	both
		Exocoetidae sp. 5	Flyingfishes	2	ND	022_Sterivex
		Exocoetidae sp. 6	Flyingfishes	2	ND	022_Sterivex
		Exocoetidae sp. 7	Flyingfishes	ND	2	045_Sterivex
		Exocoetidae sp. 8	Flyingfishes	1	ND	022_Sterivex
	Hemiramphidae	Hemiramphidae sp.	Flyingfishes	ND	1	045_Sterivex
Pleuronectiformes	Bothidae	Bothidae sp.	Lefteye flounders	ND	1	045_Sterivex
		Bothus pantherinus	Leopard flounder	ND	1	045_Sterivex
Scombriformes	Gempylidae	*Diplospinus multistriatus*	Striped escolar	1	ND	022_Sterivex
		Gempylus serpens	Snake mackerel	ND	2	045_Sterivex
	Scombridae	*Katsuwonus pelamis*	Skipjack tuna	3	4	both
	Chiasmodontidae	Chiasmodontidae sp.	Black swallowers	1	1	both
		Dysalotus sp.	Black swallowers	ND	1	045_Sterivex
	Bramidae	Bramidae sp. 1	Pomfrets	1	ND	022_Sterivex
		Scombriformes sp.	Tunas and others	ND	1	045_Sterivex
Labriformes	Labridae	*Calotomus* sp.	Wrasses	ND	1	045_Sterivex
		Thalassoma cupido	Cupid wrasse	ND	1	045_Sterivex
Perciformes	Pomacentridae	*Abudefduf* sp.	Damselfishes	1	ND	022_Sterivex
	Serranidae	*Epinephelus areolatus*	Areolate grouper	2	7	both
		Epinephelus sp.	Sea basses	ND	1	045_Sterivex
	Emmelichthyidae	Emmelichthyidae sp.	Rovers	1	4	both
Lophiiformes	Gigantactinidae	*Gigantactis* sp.	Whipnose anglers	5	6	both
Tetraodontiformes	Balistidae	*Canthidermis maculata*	Spotted oceanic triggerfish	ND	1	045_Sterivex
	Diodontidae	*Diodon* sp.	Porcupinefishes	ND	1	045_Sterivex
Total number of taxa detected				29	36	

Table S4 Comparison of taxonomic composition detected as eDNA from two different filters having different pore sizes (022_Sterivex: 0.22 µm Sterivex-GV and 045_Sterivex: 0.45 µm Sterivex-HV) at K2 in the subarctic gyre of the northwestern Pacific Ocean. The numbers in the table indicate the frequency of occurrence. ND indicates that the taxon was not detected in the sample.

Order	Family	Lowest common ancestor	Common name	022_Sterivex	045_Sterivex	Presence
Clupeiformes	Engraulidae	*Engraulis japonicus*	Japanese anchovy	5	6	both
	Clupeidae	*Sardinops sagax*	Pacific sardine	7	8	both
Salmoniformes	Salmonidae	*Oncorhynchus* sp.	Salmonids	1	2	both
Argentiniformes	Bathylagidae	*Leuroglossus schmidti*	Northern smooth-tongue	1	ND	022_Sterivex
Stomiiformes	Gonostomatidae	*Cyclothone pseudopallida*	Slender bristlemouth	ND	1	045_Sterivex
	Stomiidae	*Tactostoma macropus*	Longfin dragonfish	1	5	both
Myctophiformes	Myctophidae	*Diaphus theta*	California headlightfish	3	2	both
		Lampadena luminosa	Luminous lanternfish	ND	1	045_Sterivex
		Myctophidae sp. 1	Lanternfishes	6	8	both
		Myctophidae sp. 4	Lanternfishes	ND	1	045_Sterivex
		Myctophidae sp. 5	Lanternfishes	1	ND	022_Sterivex
		Notoscopelus japonicus	Japanese lanternfish	ND	1	045_Sterivex
		Stenobrachius leucopsarus	Northern lampfish	2	3	both
		Stenobrachius sp.	Lanternfishes	3	4	both
		Symbolophorus californiensis	Bigfin lanternfish	7	8	both
Gadiformes	Gadidae	*Gadus* sp.	Cods	1	ND	022_Sterivex
Lophiiformes	Gigantactinidae	*Gigantactis* sp.	Whipnose anglers	ND	1	045_Sterivex
Beloniformes	Exocoetidae	*Cheilopogon* sp.	Flyingfishes	ND	1	045_Sterivex
		Exocoetidae sp. 1	Flyingfishes	ND	1	045_Sterivex
	Belonidae	*Cololabis saira*	Pacific saury	ND	2	045_Sterivex
Scombriformes	Scombridae	*Katsuwonus pelamis*	Skipjack tuna	3	3	both
		Scomber sp.	Mackerels	5	7	both
		Thunnini sp.	Mackerels	1	1	both
	Bramidae	*Bramidae* sp. 2	Pomfrets	ND	1	045_Sterivex
Perciformes	Serranidae	*Epinephelus areolatus*	Areolate grouper	2	4	both
	Hexagrammidae	Hexagrammidae sp.	Greenlings	ND	1	045_Sterivex
	Cyclopteridae	Cyclopteridae sp.	Lumpfishes	1	1	both
Uranoscopiformes	Ammodytidae	*Ammodytes hexapterus*	Pacific sand lance	ND	1	045_Sterivex
Lutjaniformes	Haemulidae	*Parapristipoma trilineatum*	Chicken grunt	ND	1	045_Sterivex
Total number of taxa detected				17	26	

Table S5 Comparison of taxonomic composition detected as eDNA from two different filters having different pore sizes (022_Sterivex: 0.22 µm Sterivex-GV and 045_Sterivex: 0.45 µm Sterivex-HV) at St. 35 in the Chukchi Sea shelf. The numbers in the table indicate the frequency of occurrence. ND indicates that the taxon was not detected in the sample.

Order	Family	Lowest common ancestor	Common name	022_Sterivex	045_Sterivex	presence
Osmeriformes	Salangidae	*Mallotus villosus*	Capelin	1	ND	022_Sterivex
Gadiformes	Gadidae	*Eleginus gracilis*	Saffron cod	2	ND	022_Sterivex
		Gadidae sp.	Cods	4	2	both
		Gadus chalcogrammus	Walleye pollock	4	ND	022_Sterivex
		Gadus sp.	Cods	8	8	both
Pleuronectiformes	Pleuronectidae	Pleuronectidae sp. 1	Righteye flounders	1	3	both
		Pleuronectidae sp. 2	Righteye flounders	2	ND	022_Sterivex
		Pleuronectidae sp. 3	Righteye flounders	1	ND	022_Sterivex
Perciformes	Zoarcidae	*Gymnelus* sp.	Eelpouts	1	ND	022_Sterivex
		Lycodes raridens	Marbled eelpout	1	ND	022_Sterivex
	Stichaeidae	*Leptoclinus maculatus*	Daubed Shanny	ND	2	045_Sterivex
		Lumpeninae sp. 1	Pricklebacks	7	7	both
		Lumpeninae sp. 2	Pricklebacks	7	8	both
	Gasterosteidae	*Gasterosteus aculeatus*	Three-spined stickleback	ND	1	045_Sterivex
	Agonidae	*Ulcina olrikii*	Arctic alligatorfish	1	ND	022_Sterivex
	Cottidae	Cottidae sp.	Sculpins	7	4	both
		Gymnocanthus tricuspis	Arctic staghorn sculpin	6	5	both
		Myoxocephalus sp.	Sculpins	3	ND	022_Sterivex
	Liparidae	*Liparis gibbus*	Variegated snailfish	2	ND	022_Sterivex
Uranoscopiformes	Ammodytidae	*Ammodytes hexapterus*	Pacific sand lance	8	8	both
		Ammodytes sp.	Sand lances	8	8	both
Total number of taxa detected				19	11	

Table S6 Taxonomic composition detected as eDNA using 0.45 µm Sterivex-HV (045_Sterivex) at St. 10 in the Chukchi Sea slope. The numbers in the table indicate the frequency of occurrence. ND indicates that the taxon was not detected in the sample.

Order	Family	Lowest common ancestor	Common name	045_Sterivex
Gadiformes	Gadidae	*Boreogadus saida*	Arctic cod	7
		Eleginus gracilis	Saffron cod	1
		Gadus sp.	Cods	6
Pleuronectiformes	Pleuronectidae	Pleuronectidae sp. 1	Righteye flounders	3
Perciformes	Zoarcidae	*Lycodes palearis*	Wattled eelpout	1
		Lycodes sp.	Eelpouts	2
	Stichaeidae	*Leptoclinus maculatus*	Daubed Shanny	1
		Lumpeninae sp. 1	Pricklebacks	8
		Lumpeninae sp. 2	Pricklebacks	8
		Stichaeidae sp. 1	Pricklebacks	8
		Stichaeidae sp. 2	Pricklebacks	8
		Stichaeidae sp. 3	Pricklebacks	5
	Gasterosteidae	*Gasterosteus aculeatus*	Three-spined stickleback	1
Uranoscopiformes	Ammodytidae	*Ammodytes hexapterus*	Pacific sand lance	8
		Ammodytes sp.	Sand lances	8
Total number of taxa detected				15

Table S7 Results of permutational analysis of variance (PERMANOVA) based on the Jaccard dissimilarity to test differences in fish taxonomic composition obtained by eDNA metabarcoding between filter types (022_Sterivex: 0.22 μm Sterivex-GV and 045_Sterivex: 0.45 μm Sterivex-HV).

Site	Term	DF	Sum of squares	R^2	Pseudo-F ratio	P value
P03	Filter	1	0.497	0.136	1.883	**0.007**
	Residual	12	3.167	0.864		
	Total	13	3.664	1.000		
K2	Filter	1	0.131	0.054	0.746	0.694
	Residual	13	2.279	0.946		
	Total	14	2.410	1.000		
St35	Filter	1	0.205	0.141	2.304	**0.032**
	Residual	14	1.247	0.859		
	Total	15	1.453	1.000		

Table S8 Results of permutational dispersion test (PERMDISP) based on the Jaccard dissimilarity to test multivariate homogeneity of group dispersions in fish taxonomic composition obtained by eDNA metabarcoding grouped by filtration replicates.

Site	Term	DF	Sum of squares	Mean squares	F value	P value
P03	Filter	1	0.000838	0.000838	0.082308	0.779
	Residuals	12	0.122113	0.010176		
K2	Filter	1	0.000513	0.000513	0.060423	0.810
	Residuals	13	0.110340	0.008488		
St35	Filter	1	0.020387	0.020387	2.698941	0.123
	Residuals	14	0.105751	0.007554		
